# Self-determination theory interventions versus usual care in people with diabetes: a systematic review with meta-analysis and trial sequential analysis

**DOI:** 10.1186/s13643-023-02308-z

**Published:** 2023-09-06

**Authors:** Anne Sophie Mathiesen, Vibeke Zoffmann, Jane Lindschou, Janus Christian Jakobsen, Christian Gluud, Mette Due-Christensen, Bodil Rasmussen, Emilie Haarslev Schröder Marqvorsen, Trine Lund-Jacobsen, Tine Bruhn Skytte, Thordis Thomsen, Mette Juel Rothmann

**Affiliations:** 1 Department of Endocrinology, Center for Cancer and Organ Diseases, Copenhagen University Hospital — Rigshospitalet, Copenhagen, Denmark; 2 Steno Diabetes Center Odense, Odense University Hospital, Odense, Denmark; 3 The Interdisciplinary Research Unit of Women’s, Children’s and Families’ Health, the Julie Marie Center, Copenhagen University Hospital — Rigshospitalet, Blegdamsvej 9, Copenhagen, 2100 Denmark; 4 Sector of Health Services Research, Department of Public Health, University of Copenhagen, Copenhagen, Denmark; 5 Faculty of Health, School of Nursing and Midwifery, Deakin University, Melbourne, Australia; 6 Copenhagen Trial Unit, Centre for Clinical Intervention Research, Copenhagen University Hospital — Rigshospitalet, The Capital Region, Copenhagen, Denmark; 7 Department of Regional Health Research, The Faculty of Heath Sciences, University of Southern Denmark, Odense, Denmark; 8 Faculty of Nursing, Midwifery and Palliative Care, King’s College London, London, UK; 9 Steno Diabetes Center Copenhagen, The Capital Region of Denmark, Herlev, Denmark; 10 Department of Anaesthesiology, Department of Clinical Medicine, Herlev & Gentofte Hospital, University of Copenhagen, Copenhagen, Denmark; 11 Department of Clinical Research, Faculty of Health Science, University of Southern Denmark, Odense, Denmark

**Keywords:** Quality of life, Diabetes distress, Glycated hemoglobin, Health education tools, Psychosocial support

## Abstract

**Background:**

Autonomy-supporting interventions, such as self-determination theory and guided self-determination interventions, may improve self-management and clinical and psychosocial outcomes in people with diabetes. Such interventions have never been systematically reviewed assessing both benefits and harms and concurrently controlling the risks of random errors using trial sequential analysis methodology. This systematic review investigates the benefits and harms of self-determination theory-based interventions compared to usual care in people with diabetes.

**Methods:**

We used the Cochrane methodology. Randomized clinical trials assessing interventions theoretically based on guided self-determination or self-determination theory in any setting were eligible. A comprehensive search (latest search April 2022) was undertaken in CENTRAL, MEDLINE, Embase, LILACS, PsycINFO, SCI-EXPANDED, CINAHL, SSCI, CPCI-S, and CPCI-SSH to identify relevant trials. Two authors independently screened, extracted data, and performed risk-of-bias assessment of included trials using the Cochrane risk-of-bias tool 1.0. Our primary outcomes were quality of life, all-cause mortality, and serious adverse events. Our secondary outcomes were diabetes distress, depressive symptoms, and nonserious adverse events not considered serious. Exploratory outcomes were glycated hemoglobin and motivation (autonomy, controlled, amotivation). Outcomes were assessed at the end of the intervention (primary time point) and at maximum follow-up. The analyses were conducted using Review Manager 5.4 and Trial Sequential Analysis 0.9.5.10. Certainty of the evidence was assessed by GRADE.

**Results:**

Our search identified 5578 potentially eligible studies of which 11 randomized trials (6059 participants) were included. All trials were assessed at overall high risk of bias. We found no effect of self-determination theory-based interventions compared with usual care on quality of life (mean difference 0.00 points, 95% *CI* −4.85, 4.86, *I*^2^ = 0%; 225 participants, 3 trials, TSA-adjusted CI −11.83, 11.83), all-cause mortality, serious adverse events, diabetes distress, depressive symptoms, adverse events, glycated hemoglobulin A1c, or motivation (controlled). The certainty of the evidence was low to very low for all outcomes. We found beneficial effect on motivation (autonomous and amotivation; low certainty evidence).

**Conclusions:**

We found no effect of self-determination-based interventions on our primary or secondary outcomes. The evidence was of very low certainty.

**Systematic review registration:**

PROSPERO CRD42020181144

**Supplementary Information:**

The online version contains supplementary material available at 10.1186/s13643-023-02308-z.

## Introduction

More than 425 million people are affected by diabetes worldwide, and of these, type 2 diabetes accounts for 90% [1]. People with type 1 and 2 diabetes have to manage complex and demanding self-management tasks in their everyday life. To adequately support these tasks, diabetes care management should consider the person’s age, cognitive abilities, literacy, social, cultural factors, diabetes complications and comorbidities, health priorities, and preferences of care [2]. One way to support patient engagement and long-term improvement in diabetes care may be the use of autonomy-supporting interventions facilitating shared decision-making and collaborative goal setting. Intrinsic motivation is a key element in autonomy interventions as it is associated to successfully achieving and sustaining treatment targets [3].

Existing self-management and behavioral interventions for diabetes vary in their content, and the long-term effectiveness is uncertain [4, 5]. Several interventions including educational, psychological, and health educational tools are based on different theoretical foundations, training, and clinical skills. Meta-analyses on interventions grounded in behavioral change theory have indicated that these interventions are more effective than interventions that are not theoretically grounded [6, 7].

Meta-analyses of psychological interventions addressing emotions, cognitions, and behaviors proved non-effective for reducing glycated hemoglobin (HbA1c) in people with type 1 [8] or type 2 diabetes [4]. Health educational tools targeted translation of person-centered care into practice, and enhance intrinsic motivation may lead to greater long-term behavior change than tools solely relying on external motivation [9].

The guided self-determination intervention developed by Zoffmann [10, 11] and interventions based on self-determination theory by Deci and Ryan [12] are autonomy-supportive methods. Guided self-determination is an empowerment-based method recognized as a life-skills approach [13], and empirically developed from grounded theory [10, 11, 14, 15], and formal theories including self-determination theory and life-skills theory.

The guided self-determination method is hypothesized to improve clinical outcomes through the following pathways [15, 16]: increased perceived autonomy, a higher frequency of self-monitored blood glucose, increased perceived competence in managing diabetes, decreased diabetes-related distress, and ultimately improved glycemic control [10, 11, 14, 15].

The self-determination theory is based on comprehensive empirical research. According to the self-determination theory, enhanced autonomous motivation and mental health are met when the three basic psychological needs, competence, autonomy, and relatedness, are satisfied [12, 17]. Self-determination theory proposes a continuum for the internalization of motivation, whereby people become more autonomous (or self-determined) to engage in behaviors over time. The pathways of mechanisms behind enhanced autonomy are built on a theoretical model [18], which argues that social-contextual events (e.g., feedback, communications, rewards) that conduce towards feelings of competence during action can enhance intrinsic motivation. Accordingly, tailored feedback and lack of demeaning evaluations are hypothesized to facilitate intrinsic motivation and thereby promote autonomy.

Previous reviews including randomized trials and non-randomized studies have been carried out [3, 6, 19]; however, all three reviews [19–21] included trials from diverse populations, primarily with healthy people and multiple experimental designs. Nevertheless, whether an improvement can be attributed to the intervention, it can only be established in randomized clinical trials. A detailed overview of the characteristics of the three reviews can be found on our protocol [22]. None of the reviews had a registered or published protocol, neither were they based on unrestricted searches, and bias of risk was only assessed in two reviews applying selected domains adopted from the Cochrane Handbook [23, 24]. None of the reviews controlled the risks of random errors using trial sequential analysis.

Nevertheless, the guided self-determination method (GSD) [10, 11, 14, 15] and self-determination theory (SDT) [12] aim to enhance autonomous motivation and behavior change and may thereby improve clinical outcomes. Due to the limitations of the existing reviews and the fact that guided self-determination intervention method had not yet been systematically reviewed, we find it justified to conduct a systematic review including trial sequential analysis (TSA) and Grading of Recommendations, Assessment, Development and Evaluations (GRADE) for assessing the potential of an effect, specifically targeting people with diabetes.

### Objective

The objective was to investigate the benefits and harms of guided self-determination and self-determination theory interventions versus usual care in people with diabetes type 1 or type 2.

## Materials and methods

We conducted this systematic review according to our protocol published prior to conducting the literature searches [22]. In short, we conducted this review following Cochrane guidelines [20] and reported according to the Preferred Reporting Items for Systematic Reviews and Meta-Analysis Protocols (PRISMA-P) 2020 statement (Supplementary file 2, PRISMA-P expanded checklist 2020) [25]. The analyses were performed using the Review Manager 5.3 [26] and the TSA 0.9.5.10 beta software [27]. Deviations from the published protocol were recorded and are elaborated in the section “Differences between the protocol and the systematic review”.

### Eligibility criteria of the included trials

We included randomized clinical trials and cluster-randomized clinical trials (parallel, factorial, or crossover design) investigating interventions theoretically based on guided self-determination or self-determination theory conducted in any setting. Trials were defined as a guided self-determination trial if the reflection sheets specific for the method were applied. Trials were included irrespective of publication status, reported outcomes, publication date, publication type, and language [20]. Participants were adolescents (13 to 18 years) or adults with type 1 or type 2 diabetes. Control interventions were attention control [28], “no intervention”, wait list, or standard care as defined by trialists (e.g., standard healthcare provision).

### Information sources

#### Search strategy and electronic searches

We searched the Cochrane Central Register of Controlled Trials (CENTRAL), Medical Literature Analysis and Retrieval System Online (MEDLINE), Excerpta Medical database (Embase), Latin American and Caribbean Health Sciences Literature (LILACS), PsycINFO, Science Citation Index Expanded (SCI-EXPANDED), Cumulative Index to Nursing and Allied Health Literature (CINAHL), Social Sciences Citation Index (SSCI), Conference Proceedings Citation Index-Science (CPCI-S), and Conference Proceedings Citation Index-Social Science & Humanities (CPCI-SSH) to identify relevant trials. All databases were searched from their inception to the present. The latest literature searches were performed in April 2022 and inclusion ended in April 2022 [22]. (For a detailed search strategy for all electronic databases, see Supplementary file 1, search strategy.)

#### Selection processes

Two authors independently screened, extracted data, and performed risk-of-bias assessment of included trials using the Cochrane risk-of-bias tool. If data were missing or unclear, we contacted the trial author by email. Disagreements were solved by consulting a third author.

#### Data collection process

All potentially eligible trials identified in the literature searches were imported into the systematic review management program, Covidence [29]. Two authors (A. S. M.) and (J. L.) independently screened potentially eligible trials and subsequently extracted data from included trials. Disagreements were solved by consulting a third author (V. Z. or J. C. J.).

Trial data extracted included trial characteristics, participants characteristics, and diagnosis. Intervention group and control group characteristics, education and training of the interventionists, outcomes, funding, and conflict of interests (Table 1, characteristics of included trials). For a detailed list of trial data extracted, we refer to our protocol [22].

**Table 1:** Characteristics of included individually randomized and cluster-randomized clinical trials

**Author (year)** **Clinicaltrials.gov ID**	**Design**	**Setting, country**	**Total (*n*)**	**Intervention**	**Control group**	**Participants**	**Type of diabetes**	**Mean age**	**Mean duration of diabetes (years)**	**Deliverer**	**Outcomes**	**Follow-up time points (months)**
**Brorson et al. (2019)** [30]	A cluster-randomized controlled trial	Outpatient setting, Sweden	71	Guided self-determination (group based)	Standardized pump start education, follow-up after 1 week, follow-up after 4–6 weeks and 4 months after start	Adolescents (12–18 years)	Type 1	15.1 years (12–17.99)	NR^a^	“Guided self-determination youth group leaders (educational background not described)	Primary outcome: Hba1c Secondary outcomes: DISABKIDS, check your health, the Diabetes Family Conflict scale (DFCS), the Swedish Diabetes Empowerment Scale (Swe-DES 23), usage of continuous glucose monitoring system. Multiple daily injections	6 and 12 months
**Husted et al. (2014) **[31]	A randomized clinical trial	Outpatient setting, Denmark	71	Guided self-determination (revised for adolescents)	Eight sessions scheduled equal to the intervention group across an 8- to 12-month period, with a duration of 30 to 45 min. They received HbA1c measurement, advice on glycemic control, and insulin administration. Parents participated	Adolescents (13–18 years) HbA1c > 64 mmol/mol	Type 1	15 years	5.7 years	Two pediatric physicians, five pediatric diabetes nurses, and two dieticians (HCPs) provided the GSD-Y intervention	Perceived competence for diabetes management (PCD), Health Care Climate Questionnaire (HCCQ) assessing the degree to which patients believed their HCPs to be autonomy supportive versus Treatment Self-Regulation Questionnaire (TSRQ) assessing the motivation for diabetes management and the degree to which behaviors tended to be self-determined. Problem Areas in Diabetes (PAID) assessing diabetes-related distress including a wide range of feelings related to living with diabetes and the Perception of Parents Scale (POPS)	Intervention group 215 ± 59 days versus control group 246 ± 83 days
**Mohn et al. (2017)** [32] **ClinicalTrials. gov NCT 01317459**	A randomized clinical trial	Outpatient setting, Norway	178 (62% women)	Guided self-determination (group based)	Usual care	Adults (> 18 years old), HbA1c > 64 mmol/mol	Type 1	18–55 years, mean age 36.7 ± 10.7	19 years, range (1–46)	Diabetes specialist nurses trained in the guided self-determination method	HbA1c Self-monitored blood glucose (SMBG), PAID; DDS, PCDS, Rosenberg Self-Esteem Scale (RSES), WHO-5, HCCQ, TSRQ, treatment Relative Autonomy Index (RAI)	9 months
**Zoffmann et al. (2006)** [15]	A randomized clinical trial	Outpatient setting, Denmark	50	Guided self-determination (group based)	Delayed group training	Adults (> 18 years old), HbA1c > 64 mmol/mol	Type 1	36.8 ± 1.7 (exp.) 35.7 ± 2.1 (cont.)	Age at onset of diabetes 18.2 ± 2 (exp.) 13 ± 2.2 (cont.)	Nurses trained in the guided self-determination method	Health Care Climate Questionnaire (HCCQ) Treatment Self-Regulation Questionnaire (TSRQ) Perceived Competence in Diabetes Scale (PCD) Problem Areas in Diabetes (PAID)	12 months
**Zoffmann et al. (2015)** [16, 33] **Trial registration: ISRCTN70566290**	A randomized clinical trial	Outpatient setting, Denmark	200, balanced 2:1	Guided self-determination (group based or individual)	18-month delayed GSD intervention During the control period, participants received care as usual and met for sessions with nurse, doctor, or dieticians every 3–4 months	Adults (18 to 35 years old), HbA1c ≥ 64 mmol/mol	Type 1	25.7	13.7	Seven diabetes nurse specialists with 7–25 years of experience within the field of diabetes. They were GSD certified passing a test after 40 h of systematized training in the theoretical background and practical use of GSD, and all of them were supervised by V. Z.	Primary outcome: HbA1c Secondary outcomes: PAID, WHO-5, Rosenberg SES, Perceived Competence in Diabetes (PCD, Treatment Self-regulation Questionnaire (TSRQ), SMBG, Autonomy Index	HbA1c: 3, 6, 9, 12, 15, 18 months Secondary outcomes 9 and 18 months
**Juul et al. (2014)** [34] **ClinicalTrials.gov** i**dentifier: NCT01187069**	A cluster-randomized trial	General practice, Denmark	4035 (56.5% men)	Self-determination theory (individual)	Usual practice at their general practitioner	NR	Type 1 (15% and type 2 85%)	60.4 ± 8.6 years	8 years	A total of 34 nurses from 19 of 20 intervention practices had received the core intervention. 22 nurses from the 13 practices had completed the full course	Primary outcomes: HbA1c, total cholesterol, PAID	18 months (average)
**Mathiesen (2019)** [35]	A randomized clinical feasibility trial	Outpatient clinic setting, Denmark	20	Guided self-determination (individual)	Usual care	Adults (> 18 years old), HbA1c > 53 mmol/mol	Type 2 diabetes	58 years (mean)	NR	The guided self-determination intervention was provided by the PhD student certified in the method	Primary outcomes: HbA1c, diabetes distress, depressive symptoms, physical activity, hip/waist ratio, eating habits	4-month follow-up
**Nansel et al. (2015)** [36] **ClinicalTrials.gov** i**dentifier: NCT00999375**	A randomized clinical trial	Outpatient clinic setting, USA	136 (48.5% male)	Self-determination theory (Individual)	Received an equal amount of session and frequency of contacts with research staff and an equal frequency of 3-day masked CGM. Participants in the control group received no additional dietary advice beyond that provided as part of standard type 1 diabetes care. Scales, measuring cops, and spoons were provided to both groups	Adolescents 8–16.9 years old), HbA1c ≥ 6.5%, and ≤ 10%	Type 1	NR	6.0 years	Research assistants trained in pediatric T1 diabetes, intervention procedures, and motivational interviewing. Study investigators provided feedback to audiotaped role-play practice sessions prior to intervention delivery and on a random sample of audiotaped intervention sessions	Primary outcomes: diet quality, measured by the Healthy Eating Index 2005 (HEI2005), the whole plant food density (WPFD), measure and Hba1c	15 and 18 months
**Vanroy et al. (2017)** [23] **Clinical Trials.gov NCT 02064335**	A randomized controlled pilot trial	Belgium	48 (27 men and 21 women)	Self-determination theory (individual)	Similar intervention 6 months delayed. The participants in the control group were told that during the waiting period, their health measurements were analyzed	≥ 18 years old	Type 2 diabetes	Experimental grou*p*: 65 ± 8 Control grou*p*: 59 ± 8	NR	An intake and an outtake session with a professional PA coach, who held the degree of Master in Physical Education and Movement Sciences and who was familiarized with SDT and motivational interviewing	Hba1c and measurement of physical activity (armband SenseWear). A minimum of 3 weekdays and 1 weekend day was considered a valid measurement week	1–5 months and 6 months
**Glasgow et al. (2005)** [24]	A randomized clinical trial	Outpatient clinic setting, USA	886	Self-determination theory (individual)	Participants completed a touch screen computer assessment procedure involving the Provider Recognition Program measures as well as a general health risk appraisal items (e.g., use of seatbelts, cancer screening, etc.). They had the same number of visits as intervention patients and received a printout but one that focused on general health tasks and risk reduction that did not address the DPP	≥ 25 years old	Type 2 diabetes	Intervention 62 ± 1.4 Control 64 ± 1.3	NR	NR	Primary outcomes: self-management goalsetting medical nutrition therapy, dilated eye examination, foot examination Secondary outcomes: PAID 2, PHQ-9 depression scale, lipids and HbA1c, blood pressure, microalbuminuria	12 months
**Yun et al. (2020)** [37] **ChiCTR1900024354**	A cluster-randomized controlled trial	General practitioner, China	364	Self-determination theory (group based or individual)	2 control group interventions 1) Usual care group offering regular public health management services 2) Social support group (SSG) providing 3-month social support intervention based on problem-solving principles	≥ 18 years old	Type 2 diabetes	65.14 ± 7.23 years old	NR	Community doctors	Primary outcome: HbA1c	3 and 6 months

^a^Not reported

#### Outcomes

We assessed three primary outcomes: quality of life, all-cause mortality, and serious adverse events. Our secondary outcomes were diabetes distress, depressive symptoms, and adverse events considered non-serious. We also assessed two explorative outcomes: HbA1c and motivation (increased autonomy, decreased control, and decreased amotivation (discouraged lacking faith in own actions)). All outcomes were assessed at end of intervention (our primary follow-up time point) and at maximum follow-up. We assessed a potential effect in both random-effects and fixed-effect meta-analyses. We predefined 10 subgroup analyses for our three primary outcomes [22].

#### Unit of analysis issues

We included randomized clinical trials only [22]. For trials using crossover design, we had planned to only include data from the first period [20, 38]. Cluster-randomized trials were included after adjusting the original sample size of the trial to the effective sample size using the intracluster correlation coefficient from the “design effect” [20]. Meta-analyses including both individual and cluster-randomized trials were conducted as subgroup analyses (Supplementary file 4, results).

#### Risk-of-bias assessment

Risk of bias in included trials was assessed based on the domains described below [18, 20, 39–47]. These domains are as follows: random sequence generation, allocation concealment, blinding of participants and personnel, blinding of outcome assessment, incomplete outcome data, selective outcome reporting, and other bias.

All domains were considered at “low risk of bias”, “unclear risk of bias”, or “high risk of bias”. Detailed criteria for risk-of-bias assessment are outlined in our protocol [22]. This assessment was done in pairs by two independent review authors (A. S. M. and J. L.), separately for each outcome and comparison and ultimately considered in relation to overall reliability of the evidence. A trial was judged to be at low overall risk of bias if assessed as having low risk of bias in all of the above domains. A trial was judged to be at high overall risk of bias if assessed as having unclear or high risk of bias in one or more of the above domains.

We assessed the domains “blinding of outcome assessment”, “incomplete outcome data”, and “selective outcome reporting” for each outcome result. Thus, we assessed the bias risk for each outcome assessed in addition to each trial.

#### Assessment of statistical and clinical significance

All meta-analyses and subgroup analyses were conducted using Review Manager 5.4 [26]. TSA was used to control random errors [48]. To control the risk of systematic errors, we assessed the risk of bias of all included trials. The thresholds for statistical significance were adjusted according to our three primary, three secondary, and two explorative outcomes as suggested by Jakobsen et al. [48]. Thus, we considered a* p*-value of ≤ 0.014 as the threshold for statistical significance.

For dichotomous outcomes, we calculated risk ratios (RRs) with 95% confidence interval (CI), as well as the TSA-adjusted CIs. We calculated the mean differences (MDs) with 95% CI for continuous outcomes. It was not possible to calculate the standardized mean difference (SMD) due to lack of data.

We primarily investigated forest plots to visually assess signs of heterogeneity. Secondly, we assessed the presence of statistical heterogeneity by chi-square test (threshold *P* < 0.10) and measured the quantities of heterogeneity by the *I*^2^ statistic [21, 49]. We investigated possible heterogeneity through subgroup analyses. We found the rationale for conducting meta-analyses was justified [20].

### Synthesis methods

#### Dealing with missing data

We used intention to treat data if such data were available. As the first option, we contacted trial authors to obtain any relevant missing data (i.e., for data extraction and for assessment of risk of bias, as specified above). Secondly, we investigated the effects of missing data in sensitivity analyses (Supplementary file 3). We did not impute missing values for any outcomes in our primary analysis.

We primarily analyzed continuous outcome scores assessed at single time points. If changes from baseline scores were reported only, we analyzed the results together with the follow-up scores [20]. If standard deviations (SDs) were not reported, we calculated the SDs using trial data, if possible.

### Data synthesis

#### Meta-analysis

We conducted the meta-analyses according to the recommendations stated in the Cochrane Handbook for Systematic Reviews of Interventions [20], Keus et al. [50], and the eight-step assessment suggested by Jakobsen et al. [48]. The intervention effects were assessed with both random-effects meta-analyses [51] and fixed-effect meta-analyses [52]. We primarily reported the most conservative result (highest *p*-value) and considered the less conservative result as a sensitivity analysis [48]. We assessed a total of three primary, three secondary outcomes, and two explorative outcomes and therefore considered a *p*-value of 0.014 or less as the threshold for statistical significance [48]. For further details, we refer to our protocol [22].

We also controlled the risks of type 1 errors and type 2 errors and thereby the risk of potential false-positive findings of meta-analyses [53] using TSA 0.9.5.10 beta on all outcomes. We performed trial sequential analysis on all outcomes, in order to calculate the required information size (that is, the number of participants needed in a meta-analysis to detect or reject a certain intervention effect) and the cumulative Z curve’s breach of relevant trial sequential monitoring boundaries. For dichotomous outcomes, we planned to estimate the required information size based on the observed proportion of patients with an outcome in the control group (the cumulative proportion of patients with an event in the control groups relative to all patients in the control groups), a relative risk reduction of 20%, an alpha of 1.4% for all our outcomes, a beta of 10%, and the observed diversity as suggested by the trials in the meta-analysis. For the continuous outcomes, we used the observed SD in the trial sequential analysis, a mean difference of the observed SD/2, an alpha of 1.4% for all outcomes, a beta of 10%, and the observed diversity as suggested by the trials in the meta-analysis. A more detailed description of the TSA applied in this review can be found in the protocol [22] and in the TSA manual [54] or at http://www.ctu.dk/tsa/.

### Subgroup analysis

The following ten exploratory subgroup analyses were planned on the primary outcomes, quality of life, mortality, and serious adverse events [22]:

- Type of diabetes (trials including participants with type 1 compared to trials including participants with type 2)
- Socioeconomic status (trials including participants with low socioeconomic status compared to trials including participants with high socioeconomic status)
- Number of comorbidities
- Men compared to women
- Adolescent (13 to 18 years) compared to adults (> 18 years)
- Self-determination theory compared to trials investigating guided self-determination method
- Trials with an experimental intervention above and below the mean difference in intervention length
- Individual interventions compared to trials investigating group interventions
- Type of control intervention (no intervention, standard care, or placebo attention control)
- Trials with overall high risk of bias compared to trials with overall low risk of bias [22]

### Sensitivity analysis

To assess the potential impact of missing data, we performed the “best-worst-case” scenario and “worst-best-case” scenario analyses on both the primary and secondary outcomes (Supplementary file 3). For further details, we refer to our protocol [22].

#### Assessment of reporting bias

We were not able to assess reporting bias as planned, as none of our outcomes included more than 10 trials.

### The certainty of evidence

#### Summary of findings table

The certainty of the evidence was assessed by GRADE [20, 55, 56] using the five GRADE considerations (risk of bias, consistency, imprecision, indirectness, and publication bias) and the software GRADEpro GDT [56].

The certainty of the evidence was assessed independently by two authors (A. S. M. and J. L.) on the primary outcomes (quality of life, mortality, serious adverse events), the secondary outcomes (diabetes distress, depressive symptoms, and nonserious adverse events), and the explorative outcome (HbA1c and motivation).

## Results

**Fig. 1:**
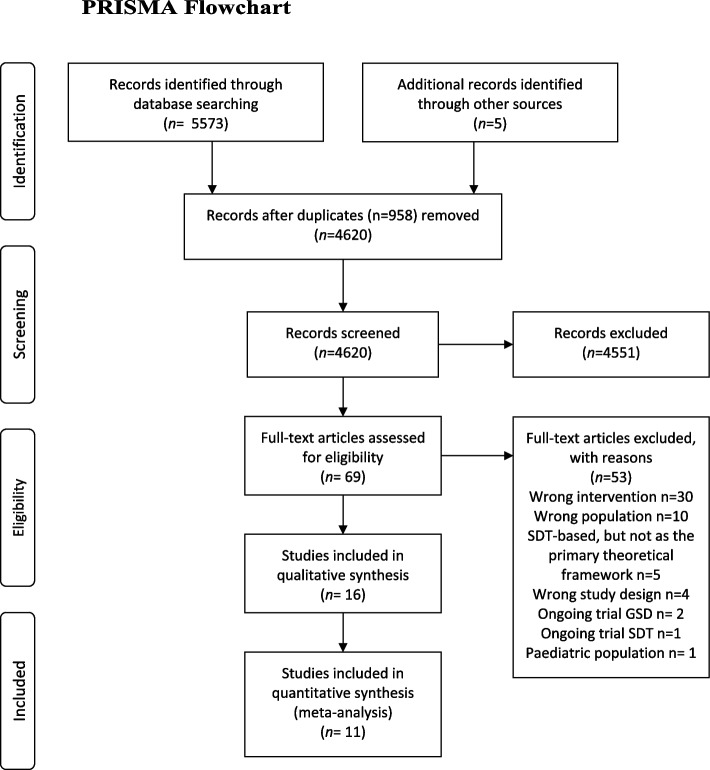
PRISMA flowchart

The initial searches yielded 5578 references of which 958 duplicates were found. Screening of title and abstracts resulted in exclusion of 4551 references. Thus, 69 references were full text screened of which 16 references representing 11 unique trials involving initial recruitment of 6059 participants were included. Trial selection and reasons for exclusion are displayed in the PRISMA flowchart (Fig. 1) [57]. Sample sizes ranged from 20 to 4034 participants [34, 35]. Five trials provided a self-determination theory-based intervention [23, 24, 34, 36, 37], and six provided a guided self-determination intervention [15, 32, 33, 35] or a GSD version revised for adolescents [30, 58] (Table 1, Characteristics of included trials). Five trials originated from Denmark [15, 33–35, 58], two originated from the USA [24, 36], and one each from Sweden [30], Norway [32], China [37], and Belgium [23]. We included four cluster-randomized trials [24, 30, 34, 37] and adjusted the original sample size of the trial to the effective sample size using the intracluster correlation coefficient from the “design effect” [20]. Brorson et al. [30] did not provide the number of clusters, which meant that we were unable to calculate the effective sample size and include data from this trial. We contacted seven trial authors by email to specify any missing data, but only one provided the requested data. We contacted three authors who had ongoing trials registered in trial registers; none of them replied.

### Risk of bias

All included trials were adjudicated at high risk of bias on minimum 2 domains. For “risk of bias” for the individual outcomes, we refer to the “Primary outcomes” section below. For risk of bias on the individual trials, the risk of bias is displayed in the meta-analyses.

### Primary outcomes

#### Quality of life: end of intervention

**Fig. 2:**
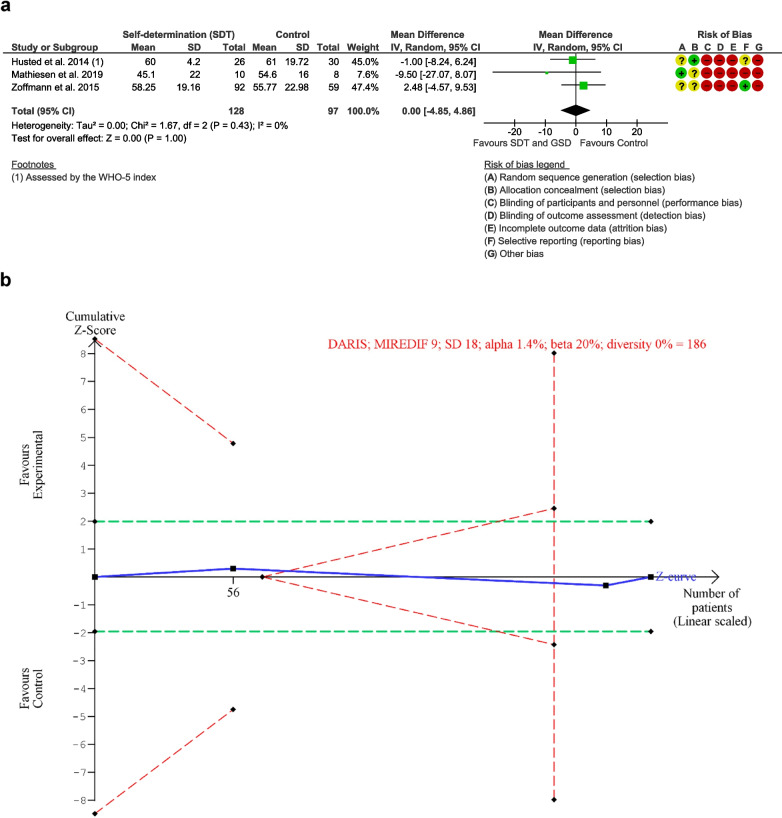
Meta-analysis and trial sequential analysis (TSA) for quality of life, end of intervention for self-determination theory vs. control. **a** Meta-analysis. **b** TSA. The diversity-adjusted required information size (DARIS) was calculated according to a mean difference of 9 points, which is half of the observed SD of 18, alpha of 1.4%, beta of 20% (80% power), and diversity 0%. The DARIS was 186 participants. The cumulative Z-curve (blue line) breaches the boundary of futility (dotted outward sloping red lines) and the DARIS. The green dotted lines show naive conventional boundaries (alpha 5%)

Three trials [33, 35, 58] including 225 participants assessed quality of life with the WHO-5 questionnaire at the end of intervention, while Brorson et al. [30] reported quality of life with the “Check your Health” questionnaire without providing data suitable for inclusion in the meta-analysis. Brorson et al. [30] did not provide the number of clusters, which meant that we were unable to calculate the effective sample size and include data from this trial. The WHO-5 ranges from 0 to 25; lower scores indicate poorer quality of life. The meta-analysis of the three trials [33, 35, 58] showed no difference between the intervention and control group on quality of life (*MD* 0.00 points, 95% *CI* −4.85, 4.86, *p* = 1.0, *I*^2^ = 0%; 225 participants, 3 trials, TSA-adjusted *CI* −11.83, 11.83). TSA showed that we had enough information to reject that self-determination theory-based intervention increased quality of life with 9 points (the diversity-adjusted required information size (DARIS) 186 participants) (Fig. 2, meta-analysis and TSA of quality of life, end of intervention). The “best-worst case” and “worst-best case” scenarios showed that missing data alone had the potential to bias the results (Supplementary material 3, sensitivity analyses). This result was at very low certainty due to serious risk of bias, serious inconsistency, and serious indirectness (Table 2, Summary of findings).

**Table 2:** Summary of findings table with GRADE evaluation for self-determination interventions compared with usual care (follow-up timepoint: end of intervention)

**Certainty assessment**							**No. of patients**		**Effect**	**Certainty**	**Importance**
No. of studies	Study design	Risk of bias	Inconsistency	Indirectness	Imprecision	Other considerations	Self-determination theory	Usual care	Relative (95% CI)		
**Quality of life** 3	RCTs	Serious^a^	Serious^b^	Serious^c^	Not serious	None	128	97	*MD* 0 (−4.85, 4.86)	⨁◯◯◯ Very low	Critical
**All-course mortality** 1	Cluster RCTs	Very serious^d^	Not serious	Serious^e^	Serious^f^	None	2005	2021	*RR* 1.13 (0.73, 1.74)	⨁◯◯◯ Very low	Critical
**SAEs** 0	-	-	-	-	-	-	-	-	-		Critical
**Diabetes distress** 3	RCTs	Serious^g^	Serious^h^	Not serious	Not serious	None	128	96	*MD* −0.10 (−6.17, 5.97)	⨁⨁◯◯ Low	Important
**Depressive symptoms** 2	RCTs	Very serious^i^	Serious^j^	Not serious	Serious^k^	None	10	10	*MD* −3.0 (−3.75, 9.74)	⨁◯◯◯ Very low	Important
**Adverse events** 0	**-**	-	-	-	-	-	-	-	-	-	Important

*RCTs* randomized clinical trials, *SAEs* serious adverse events, *CI* confidence interval, *MD* mean difference

Explanations:

^a^The three trials reporting on quality of life at the end of intervention were all rated as high risk of bias on the domains: “blinded outcome assessment” and “incomplete outcome data”

^b^Inconsistency regarding the direction of effect of included trials

^c^Downgraded due to indirectness caused by Mathiesen et al. including elderly persons with type 2 diabetes and Zoffmann et al. (2015) [16, 33] including young people with type 1 diabetes, and Husted et al. include adolescents. The three trials also apply slightly diverse versions of the guided self-determination intervention

^d^This outcome was rated as high risk of bias on the domain “blinded outcome assessor” as the first, and the last author analyzed the data in the trial. On the domain “incomplete outcome data,” it was unclear whether there where participants lost to follow-up on this domain

^e^The nurses were trained in advanced communication techniques, but the reflection sheets in the guided self-determination method were not provided to the patients

^f^TSA showed lack of data because only 3.99% of optimal information size had been reached

^g^All trials have a minimum of three high risk-of-bias domains

^h^Downgraded due to heterogeneity of the included populations (type 1 diabetes) in the trials of Zoffmann (2015) and Husted et al. (2014) [31] and type 2 diabetes in the trial of Mathiesen et al. (2019) [59]

^i^Downgraded due to “high risk of bias on blinded outcome assessor,” “incomplete outcome data,” “and selective reporting” on this outcome (only data from Mathiesen (2019) [35])

^j^Downgraded due to heterogeneity of the provided interventions

^k^Wide confidence intervals in the trial of Mathiesen et al. (2019) [59]

#### Quality of life: longest follow-up

Three trials including 335 participants assessed quality of life assessed with the WHO-5 scale at longest follow-up [32, 33, 58], while Brorson et al. [30] reported quality of life with the “Check your Health” questionnaire and did not provide data suitable for inclusion in the meta-analysis. Brorson et al. [30] did not provide the number of clusters, which meant that we were unable to calculate the effective sample size and include data from this trial. The meta-analysis of the three trials showed no difference between the intervention and the control group on quality of life (*MD* 2.82 points, 95% *CI* −2.74, 8.38, *p* = 0.32, *I*^2^ = 33%; 335 participants, 3 trials, TSA-adjusted *CI* −5.80, 11.43) (Supplementary file 4, results). TSA showed that we had enough information to reject that self-determination theory-based intervention increased quality of life with 10 points (DARIS 294 participants). The “best-worst case” and “worst-best case” scenarios showed that missing data alone had the potential to bias the results. This outcome result was overall assessed at high risk of bias as all three trials were judged to be of “high risk of bias” on the outcome domains “blinded outcome assessment”, and “incomplete outcome data” (Supplementary material 3, sensitivity analyses). The evidence was rated at very low certainty due to serious risk of bias, serious inconsistency, and serious indirectness.

#### All-cause mortality

One cluster-randomized trial assessed mortality at longest follow-up [34]. The trial showed no difference between the intervention and the control group on all-cause mortality (*RR* 1.13, 95% *CI* 0.73; 1.74, *p* = 0.59; 1529 participants (design-adjusted participant number), 1 trial). TSA could not be shown due to too little information (only 3.99% of DARIS) (Supplementary file 4, results). The result was overall assessed at high risk of bias on the domain “blinded outcome assessor” as the first and the last author analyzed the data in the trial. On the domain “incomplete outcome data”, it was unclear whether there were participants lost to follow-up. Regarding the domain “selective outcome reporting”, it was judged at low risk of bias due to the register-based design. The evidence was rated at very low certainty due to very serious risk of bias, serious indirectness, and serious imprecision (Table 2, Summary of findings).

#### Serious adverse events

None of the included trials reported serious adverse events as an outcome. Mohn et al. [32] reported that one participant dropped out in the intervention group due to referral to psychiatric care, and one dropped out from the control group due to “critical illness”. Brorson et al. [30] reported that one participant dropped out in the control group due to extremely high HbA1c values (113 mmol/mol) at 6-month follow-up [30]. This outcome result was overall assessed at high risk of bias as the two trials were judged to be of “high risk of bias” or “unclear” on the outcome domains “blinded outcome assessment”, and “incomplete outcome data”. Data was not suitable for meta-analysis (Supplementary file 5, serious adverse events and adverse events).

### Secondary outcomes

#### Diabetes distress: end of intervention

Three individually randomized trials [31, 33, 35] including 224 participants assessed diabetes distress with the PAID score at the end of the intervention. The PAID score ranges from 0 to 100; lower scores indicate less diabetes distress. The meta-analysis showed no difference between the intervention and the control group on diabetes distress (*MD* −2.59 points, −8.16, 2.98, *p* = 0.36, *I*^2^ = 0%; 224 participants, 3 trials). One cluster-randomized trial [24] reported a *MD* 1.82 points, 95% *CI* 1.69, 1.95, *p* < 0.00001, 467 participants (design effect-adjusted participant number), and 1 trial, favoring the control group (Supplementary file 4, results). The cluster-randomized trial of Glasgow et al. [24] contributed with more than 99% weight in the meta-analyses on diabetes distress. For this trial, we identified several methodological issues. First, the trial was not adequately registered in a trial register, nor was a protocol published. As such, it was not clear whether the outcomes were predefined. Furthermore, randomization, blinding, and attrition were inadequately described [24]. The TSA figure was not shown as number of included participants exceeded 100% of the DARIS. The “best-worst case” and “worst-best case” scenarios showed that incomplete data alone had the potential to influence the results (Supplementary file 3, sensitivity analyses). This result was rated at low certainty due to high risk of bias and serious inconsistency (Table 2, Summary of findings).

#### Diabetes distress: longest follow-up

**Fig. 3:**
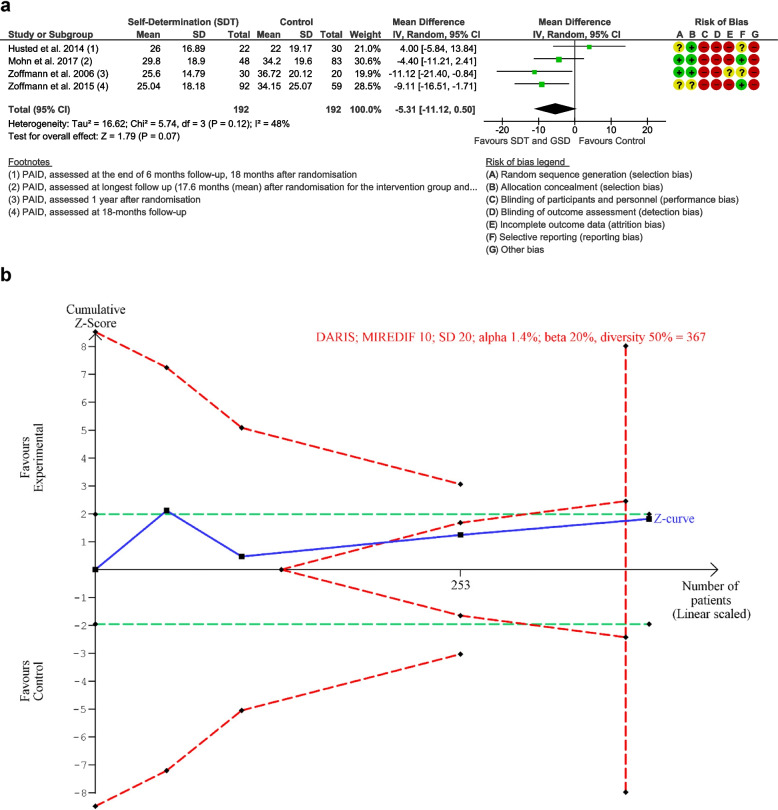
Meta-analysis and trial sequential analysis (TSA) for diabetes distress, longest follow-up for self-determination theory vs. control. **a** Meta-analysis. **b** TSA. The diversity-adjusted required information size (DARIS) was calculated according to a mean difference of 10 points, which is half of the observed SD of 20, alpha of 1.4%, a beta of 20% (80% power), and diversity 50%. The DARIS was 367 participants. The cumulative Z-curve (blue line) breaches the boundary of futility (dotted outward sloping red lines) and the DARIS. The green dotted lines show naive conventional boundaries (alpha 5%)

Four trials [15, 31–33] including 384 participants assessed diabetes distress at longest follow-up. The meta-analysis showed no difference between the intervention and control group (*MD* −5.31 points, 95% *CI* −11.12, 0.50, *p* = 0.07, I = 48%, 384 participants, four trials, TSA-adjusted *CI* −14.34, 3.72) (Supplementary file 4, results). The relatively large heterogeneity was explained by the trial of Husted et al. [31] reporting that the guided self-determination adjusted for adolescents and increased diabetes distress in young people (mean age 15 years, range 13–18 years) and relatively short duration of diabetes (mean 5.7 years). Removing the trial of Husted et al. from the forest plot reduced the heterogeneity to 0%. TSA showed that we had enough information to reject that self-determination theory-based intervention decreased diabetes distress with 10 points (DARIS 367 participants) (Figure 3, meta-analysis and TSA of diabetes distress, longest follow-up). The “best-worst case” and “worst-best case” scenarios showed that missing data alone had the potential to bias the results (supplementary material 3, sensitivity analyses). The outcome result was overall assessed at high risk of bias as all three trials were judged to be at “high risk of bias” on the domains “blinded outcome assessment” and “incomplete outcome data”. The evidence was rated at low certainty due to serious risk of bias and serious inconsistency.

#### Depressive symptoms: end of intervention

Two trials [24, 35] assessed depressive symptoms at the end of intervention. Glasgow et al. [24] reported depressive symptoms measured by the Patient Health Questionnaire (PHQ-9) but in percent, and the authors did not reply to our request for additional data. Mathiesen et al. (2019) measured depressive symptoms by the Hospital Anxiety and Depression Score (HADS) at the end of intervention and reported no difference between the intervention and the control group (*MD* −0.10 points, 95% *CI* −6.17, 5.97, *p* = 0.97) in a high risk-of-bias small feasibility trial (*n* = 20) [35]. This outcome result was overall assessed as a high risk of bias, and the evidence was rated at very low certainty due to very serious risk of bias, serious inconsistency, and serious imprecision (Table 2, Summary of findings). No trials reported on depressive symptoms at longest follow-up.

#### Adverse events: not considered serious

Mathiesen et al. (2019) reported that one participant in the intervention group experienced relapse in her paranoid schizophrenia during the intervention [35]. Data was not suitable for meta-analyses (Supplementary file 5, serious adverse events and adverse events). The outcome result was overall assessed at high risk of bias as both trials were judged to be of unclear or “high risk of bias” on the domains “blinded outcome assessment” and “incomplete outcome data” and high risk of bias on “selective reporting”. No adverse events were reported at longest follow-up.

### Exploratory outcomes

#### HbA1c: end of intervention

Four trials including 401 participants assessed HbA1c at the end of the intervention [31, 33, 35, 37]. Meta-analysis of the individually randomized trials showed no difference between the intervention and the control group (*MD* −0.07 mmol/mol, 95% *CI* −3.60, 3.46, *p* = 0.97, *I*^*2*^ = 0%; 275 participants, 3 trials, TSA-adjusted *CI* −5.62, 1.93). One cluster-randomized trial [37] reported an effect (*MD* −4.63 mmol/mol, 95% *CI* −7.49, −1.77, *p* = 0.001, 126 participants (design effect-adjusted participant number), 1 trial) (Supplementary file 4, results), favoring the intervention group. TSA of the individually randomized trials and the cluster-randomized trial showed that we had enough information to reject that self-determination theory-based intervention decreased HbA1c with 7 mmol/mol (DARIS 324 participants). This outcome result was overall assessed as a high risk of bias, and the evidence was rated at low certainty due to very serious risk of bias and serious inconsistency.

#### HbA1c: longest follow-up

Five trials [30, 32–34, 58] including 1913 participants assessed HbA1c at longest follow-up. Meta-analysis of the three individually randomized trials [32, 33, 58] showed no effect (*MD *−3.19 mmol/mol, 95% *CI *−6.22, −0.16, *p* = 0.04, *I*^2^ = 0%; 384 participants, 3 trials, TSA-adjusted *CI *−12.31, 8.90). One cluster-randomized trial reported no effect (*MD *−0.40 mmol/mol, 95% *CI *−2.20, 1.40, *p* = 0.66, 1529 participants (design-adjusted participant number), 1 trial) (Supplementary file 4, results) [34]. Brorson et al. [30] reported on Hba1c, but did not provide the number of clusters; thus, we were unable to adjust for the design effect. TSA of the individually randomized trials and the cluster-randomized trial showed that we had enough information to reject that self-determination theory-based intervention decreased HbA1c with 7 mmol/mol (DARIS 748 participants). This outcome result was overall assessed at high risk of bias as all three trials were judged to be at “high risk of bias” on the outcome domains “blinded outcome assessment”, and two trials [32, 58] had high risk of bias on the domain “incomplete outcome data”. The evidence was rated at low certainty due to very serious risk of bias and serious inconsistency.

#### Motivation (autonomy): end of intervention

Two trials including 207 participants assessed autonomy at the end of intervention [33, 58]. The meta-analysis of the two trials showed a *MD* 0.42 points, 95% *CI* 0.16, 0.67, *p* = 0.001, *I*^2^ = 0%; 207 participants, 2 trials, TSA-adjusted *CI *−0.99, 0.14 (Supplementary file 4, results) favoring the intervention group. The treatment self-regulation score (TSRS) (autonomy) ranges from 1 to 7; lower scores indicate less autonomous motivation. TSA showed that we had enough information to show that self-determination theory-based intervention increased autonomy with 0.45 points (DARIS 186 participants). This outcome result was overall assessed as a high risk of bias, and the evidence was rated at very low certainty due to very serious risk of bias.

#### Motivation (autonomy): longest follow-up

Five trials including 1248 participants assessed autonomy at longest follow-up [15, 31–34]. The meta-analysis of the four individually randomized trials [15, 32, 33, 58] showed a difference between the intervention and the control group (*MD* of 0.30 points, 95% *CI* 0.11, 0.48, *p* = 0.002, *I*^2^ = 0%; 384 participants, 4 trials, TSA-adjusted *CI *−0.01, 0.45) (Supplementary file 4, results) favoring the intervention group. One cluster-randomized trial (design-adjusted participant number) reported no effect. TSA of the individually randomized trials and the cluster-randomized trial showed that we had enough information to reject that self-determination theory-based intervention increased autonomy with 0.45 points (DARIS 557 participants).

This outcome result was overall assessed as a high risk of bias as all four included trials were judged to be of “high risk of bias” on the outcome domains “blinded outcome assessment” and “incomplete outcome data”. The evidence was rated at low certainty due to serious risk of bias and serious indirectness.

#### Motivation (control): end of intervention

Two trials including 207 participants [33, 58] assessed motivation (control) at the end of intervention. The meta-analyses of the two trials showed no difference between the intervention and the control group (0.06 points, 95% *CI *−0.26, 0.39, *p* = 0.71, *I*^2^ = 0%; 207 participants, 2 trials, TSA-adjusted *CI *−0.71, 0.83) (Supplementary file 4, results). The treatment self-regulation score (TSRS) (control) ranges from 1 to 7; lower scores indicate less controlled motivation. TSA showed that we had enough information to reject that self-determination theory-based intervention increased motivation (control) with 0.6 points (DARIS 175 participants). This outcome result was overall assessed as at high risk of bias, and the evidence was rated at low certainty due to very serious risk of bias and serious inconsistency.

#### Motivation (control): longest follow-up

Four individually randomized trials [15, 32, 33, 58] including 384 participants assessed motivation (control) at longest follow-up. Meta-analysis of the four trials showed no difference between the intervention and the control group (*MD* 0.05 points, 95% *CI *−0.19, 0.30, *p* = 0.67, *I*^2^ = 0%; 384 participants, 4 trials, TSA-adjusted *CI *−0.92, 1.07) (Supplementary file 4, results). One cluster-randomized trial (design-adjusted participant number) reported no effect. TSA showed that we had enough information to reject that self-determination theory-based intervention decreased motivation (control) with 0.6 points (DARIS 575 participants) (Supplementary file 4, results). This outcome result was overall assessed as at high risk of bias as all four included trials were judged to be of “high risk of bias” on the outcome domains “blinded outcome assessment” and “incomplete outcome data”. The evidence was rated at low certainty due to serious risk of bias and serious indirectness.

#### Motivation (amotivation): end of intervention

Two trials including 207 participants assessed motivation (amotivation) at the end of intervention. Meta-analysis of the two trials showed a difference between the intervention and the control group (*MD *−0.37 points, 95% *CI *−0.67, −0.07, *p* = 0.02, *I*^2^ = 0%; 207 participants, 2 trials, TSA-adjusted *CI*: −1.08, 0.34) favoring the intervention group (Supplementary file 4, results). The treatment self-regulation score (TSRS) (amotivation) ranges from 1 to 7; lower scores indicate less amotivation. TSA showed that we had enough information to reject that self-determination theory-based intervention decreased amotivation (DARIS 176 participants). This outcome result was overall assessed as at high risk of bias, and the evidence was rated at low certainty due to very serious risk of bias.

#### Motivation (amotivation): longest follow-up

Three trials [15, 31, 33] including 258 participants assessed amotivation at longest follow-up. Meta-analysis of the three trials showed a difference between the intervention and the control group (*MD *−0.53 points, 95% *CI *−0.62, −0.45, *p* < 0.00001, *I*^2^ = 27%; 253 participants, 3 trials, TSA-adjusted *CI*: −0.73, −0.32) favoring the intervention group (Supplementary file 4, results). TSA showed that we had enough information to show that self-determination theory-based intervention decreased amotivation with 0.2 points (DARIS 207 participants). This outcome result was overall assessed at high risk of bias as all three included trials were judged to be of “high risk of bias” on the outcome domains “blinded outcome assessment” and “incomplete outcome data”. The evidence was rated as at low certainty due to serious risk of bias and serious indirectness.

### Subgroup analysis

We predefined ten exploratory subgroup analyses on the primary outcomes, quality of life, mortality, and serious adverse events [22]. Of these, we were only able to conduct subgroup analyses on quality of life and on diabetes distress (post hoc).

When assessing quality of life, test for subgroup difference showed no evidence of a difference when comparing type of diabetes (*p* = 0.17), sex (men compared to women) (*p* = 0.12), age (adolescents compared to adults) (*p* = 0.12), length of intervention (*p* = 0.14), type of therapy (individual compared to group) (*p* = 0.06), or type of control intervention (standard care compared to waitlist design compared to attention control) (*p* = 0.23) (Supplementary file 6, subgroup analyses quality of life).

One secondary outcome (diabetes distress) was post hoc analyzed due to clinical relevance [60]. When assessing diabetes distress, test for subgroup difference showed evidence of a difference when comparing type of diabetes (*p* = 0.02) with no effect of the experimental intervention in participants with type 1 diabetes and a negative effect in participants with type 2 diabetes, guided self-determination method compared to self-determination theory-based interventions (*p* = 0.007) showing benefits of guided self-determination and harms of self-determination theory intervention, and type of therapy (individual compared to group) (*p* = 0.004) showing harms of individual therapy compared to benefits of group therapy.

We found no evidence of a difference when comparing adolescents to adults (*p* = 0.15), length of intervention (*p* = 0.15), or type of control intervention (no intervention compared to standard care compared to placebo compared to attention control) (*p* = 0.05) (Supplementary file 7, subgroup analyses diabetes distress).

We were not able to conduct any of the remaining pre-defined subgroup analyses [22] due to lack of relevant data.

### The certainty of evidence

#### Summary of findings table

Two authors (A. S. M. and J. L.) independently assessed the certainty of the evidence using the five GRADE considerations (risk of bias, consistency, imprecision, indirectness, and publication bias) and the software GRADEpro GDT [56].

The certainty of the evidence was assessed on the primary outcomes (quality of life, mortality, serious adverse events), the secondary outcomes (diabetes distress, depressive symptoms, and non-serious adverse events), and the explorative outcome (HbA1c and motivation).

The certainty of the evidence was very low on the outcomes quality of life, mortality, serious adverse events, diabetes distress, depressive symptoms, serious adverse events, and motivation (control) (Table 2, summary of findings). On the outcomes motivation (autonomy) and motivation (amotivation), the quality of the evidence was rated as “low”.

We assessed imprecision using TSA and found that imprecision was present on the outcome “all-cause mortality” (Table 2, summary of findings). We reported all decisions to downgrade the quality of the trials by footnotes to add to the transparency of the decisions.

### Differences between the protocol and the review

We were not able to report on all predefined subgroup analyses [22], as the included trials did not investigate, report, or provide the missing data on our requests. We performed post hoc subgroup analyses on our three secondary outcomes due to unexplainable heterogeneity. We needed to retrospectively report on motivation measured by the treatment self-regulation questionnaire. These changes have been submitted to PROSPERO. Basing our summary of findings table of trials assessed as low risk of bias was not an option as all included trials were at high risk of bias.

## Discussion

This systematic review synthesized the evidence for beneficial and harmful effects of guided self-determination or self-determination theory interventions for people with diabetes in any healthcare setting assessed in randomized clinical trials.

We adhered to our pre-published protocol [22] and considered both risks of random errors and risks of systematic errors by applying the Cochrane methodology [20], the eight-step assessment suggested by Jakobsen et al. [48], trial sequential analysis [27], and GRADE assessments [56]. We found no effects of self-determination theory-based interventions compared with usual care on quality of life, all-course mortality, serious adverse events, diabetes distress, depressive symptoms, adverse events, HbA1c, and motivation (controlled); all results were at very low certainty, except for diabetes distress, which was at low certainty of the evidence. We found a potential effect on motivation (autonomous and amotivation) but at low certainty of the evidence.

We found no effect on the quality of life assessed with the WHO-5 index. If these interventions have any effects on quality of life, we may need a more specific instrument than the generic WHO-5 index to assess it [61]. Also, it may be so that guided self-determination or self-determination theory interventions simply do not affect quality of life. A randomized trial testing an emotional writing intervention in people with type 2 diabetes found a potentially clinically important worsening in depressive symptoms [62]. First, this emphasizes the need of assessing harms in all trials investigating psychosocial interventions. Second, in trials that reported no benefit of the guided self-determination on quality of life, the included participants were older and more likely to have well-developed writing skills [32, 33, 35].

We were not able to show any effects on the secondary outcome diabetes distress. This might mirror the pooling of the two potentially different interventions (guided self-determination and self-determination theory). The cluster-randomized trial of Glasgow et al. [24] investigating a self-determination theory intervention found harmful effect on diabetes distress. The trial contributed with more than 99% weight in the meta-analyses on diabetes distress. This trial had several methodological issues and thus high risk of bias: the trial was not registered in a trial register, nor was a protocol published; randomization, blinding, and attrition were inadequately described [24]. The guided self-determination method makes use of the reflection sheets as a pragmatic tool for internalizing the method, which might make a difference in clinical practice. This is supported by the high heterogeneity in the analysis of diabetes distress (end of intervention) and our subgroup analyses comparing the effect of guided self-determination to self-determination theory on diabetes distress. This difference should be cautiously interpreted. However, it might indicate that the guided self-determination is more useful in problem-solving of diabetes-specific challenges and potentially a more adequate tool for reducing diabetes distress, perhaps due to the reflection sheets. Moreover, the group format enables sharing of experiences between people living with diabetes which has been shown to increase normalization of emotional challenges related to diabetes and in turn reduction of diabetes distress [63, 64]. It might also be mediated by the increase of motivation (autonomy) and decrease on amotivation found in the meta-analyses solely including trials applying guided self-determination.

We found no effects on all-cause mortality, serious adverse events, and nonserious adverse events. These outcomes were seriously underreported in the included trials. Therefore, we do not know if guided self-determination or self-determination theory interventions have any effects on these important patient related outcomes.

Regarding the secondary outcome “depressive symptoms”, our results were also prone to missing data. The evidence for all primary and secondary outcomes were rated at very low certainty, and more high-quality trials are severely needed prior to implementing interventions applying guided self-determination or self-determination theory for diabetes into clinical practice.

Subgroup analyses on diabetes distress indicated differences in effects when comparing type of diabetes, favoring type 1 diabetes, and, as mentioned on guided self-determination method compared to self-determination theory-based interventions, favoring guided self-determination, and on type of therapy (individual compared to group), favoring group therapy. However, subgroup analyses are only hypotheses generating due to a high risk of type 1 error, and these subgroup effects may be investigated further in high-quality randomized trials.

It might be questioned if the half SDs derived from the meta-analyses and applied in the TSAs always reflect the minimal clinical important difference. In our currently ongoing trial investigating guided self-determination method in people with type 2 diabetes [60], we estimated a reduction of 6 points on the Problem Areas in Diabetes scale (PAID) as the minimal clinical important difference [59]. In this systematic review, we cannot reject that self-determination theory-based intervention decreases diabetes distress with less than 10.5 point as assessed in the TSA.

A preplanned outcome focusing on diabetes self-management skills, e.g., self-monitored blood glucose, would potentially have strengthened the clinical relevance of this systematic review; however, increased autonomy, alleviation of diabetes distress, and depressive symptoms have been reported to be associated with improved diabetes self-management skills [15, 33, 65].

Overall, large attrition [32] and incomplete outcome data [24, 31, 33] leading to high risk of bias rating on this domain seemed to be a general problem associated with the trials included in this systematic review which also may overestimate effect sizes [47]. Qualitative methods [66] or realist evaluation methodology [67] may be appropriate to investigate the pathways leading to the large attrition in some trials. A general upgrading of the clinical trial management skills invested in non-pharmacological trials may improve trial methodology. The trials that we included had difficulties with blinding personnel and participants and used several subjective patient-reported outcomes.

### Limitations

Our systematic review has some important limitations. The primary limitation was the clinical heterogeneity caused by the inclusion of both types of interventions (guided self-determination and self-determination theory), both type 1 and type 2 diabetes, and all modes of delivery and interventionists. Furthermore, flaws in trial design may overestimate effect estimates and increase between-trials heterogeneity [47], especially prone to trials with subjective outcome reporting [47]. Moreover, diabetes management always consists of multiple treatment elements [2], and it is likely that different co-interventions and spill-over effects from clinical practice may also have influenced results.

We were not able to conduct most of our planned subgroup analyses due to the fact that the trials were not reporting on the predefined characteristics. Another limitation of our review was the large number of comparisons which increases the risk of type 1 error. Likewise, our meta-analyses were compromised by missing data, despite preplanned attempt to contact all authors of included trials. A further limitation was the exclusion of quasi-randomized studies and observational studies in the assessments of adverse events. By focusing on randomized clinical trials that are unlikely to identify late and rare adverse events, we run the risks of focusing too much on benefits and too little on harms. A future systematic review focusing on the risks of harms in quasi-randomized studies and observational studies should take this into account to achieve a more balanced evaluation of benefits and harms once we have demonstrated convincing benefits of the interventions.

## Conclusions

We found no effect of self-determination theory-based interventions compared with usual care on our primary outcomes: quality of life, all-cause mortality, and serious adverse events or secondary outcomes: diabetes distress, depressive symptoms, and adverse events. The evidence was of low to very low certainty.

### Supplementary Information

**Additional file 1.** Search strategies.

**Additional file 2.** PRISMA 2020 Checklist.

**Additional file 3.** Sensitivity analyses best-worst and worst-best scenarios Quality of life and diabetes distress.

**Additional file 4.** Results all outcomes.

**Additional file 5.** Serious adverse events (SAE) and Adverse events.

**Additional file 6.** Subgroup analyses, Quality of life.

**Additional file 7.** Subgroup analyses, Diabetes distress.

## Data Availability

The datasets supporting the conclusions of this article are included within the article (and its additional files).

## Abbreviations

CI: Confidence interval
DARIS: The diversity-adjusted required information size
GRADE: Grading of Recommendations, Assessment, Development and Evaluations
HADS: Hospital Anxiety and Depression Scale
HbA1c: Glycated hemoglobin
MD: Mean difference
PAID: Problem areas in diabetes
PHQ: Patient Health Questionnaire
SMD: Standardized mean difference
TSA: Trial sequential analysis
TSRS: Treatment self-regulation score
RR: Relative risk

## References

[1] Karuranga S, Fernandes JR, Huang Y, Malanda B. IDF diabetes atlas. International Diabetes Federation; 2017. https://diabetesatlas.org/upload/resources/previous/files/8/IDF_DA_8e-EN-final.pdf.

[2] Davies MJ, D’Alessio DA, Fradkin J, Kernan WN, Mathieu C, Mingrone G (2018). Management of hyperglycaemia in type 2 diabetes, 2018. A consensus report by the American Diabetes Association (ADA) and the European Association for the Study of Diabetes (EASD). Diabetologia.

[3] Ntoumanis N, Ng JYY, Prestwich A, Quested E, Hancox JE, Thogersen-Ntoumani C (2021). A meta-analysis of self-determination theory-informed intervention studies in the health domain: effects on motivation, health behavior, physical, and psychological health. Health Psychol Rev.

[4] Chew BH, Vos RC, Metzendorf MI, Scholten RJ, Rutten GE (2017). Psychological interventions for diabetes-related distress in adults with type 2 diabetes mellitus. Cochrane Database Syst Rev.

[5] Dombrowski SU, Knittle K, Avenell A, Araujo-Soares V, Sniehotta FF (2014). Long term maintenance of weight loss with non-surgical interventions in obese adults: systematic review and meta-analyses of randomised controlled trials. BMJ.

[6] Gillison FB, Rouse P, Standage M, Sebire SJ, Ryan RM (2019). A meta-analysis of techniques to promote motivation for health behaviour change from a self-determination theory perspective. Health Psychol Rev.

[7] Prestwich A, Sniehotta FF, Whittington C, Dombrowski SU, Rogers L, Michie S (2014). Does theory influence the effectiveness of health behavior interventions? Meta-analysis. Health Psychol.

[8] Winkley K, Upsher R, Stahl D, Pollard D, Brennan A, Heller S (2020). Systematic review and meta-analysis of randomized controlled trials of psychological interventions to improve glycaemic control in children and adults with type 1 diabetes. Diabet Med.

[9] Phillips AS, Guarnaccia CA. Self-determination theory and motivational interviewing interventions for type 2 diabetes prevention and treatment: a systematic review. J Health Psychol. 2017;25(1):44–66. 10.1177/1359105317737606 29119829

[10] Zoffmann V, Harder I, Kirkevold M (2008). A person-centered communication and reflection model: sharing decision-making in chronic care. Qual Health Res.

[11] Zoffmann V, Kirkevold M (2007). Relationships and their potential for change developed in difficult type 1 diabetes. Qual Health Res.

[12] Ryan RM, Deci EL (2000). Self-determination theory and the facilitation of intrinsic motivation, social development, and well-being. Am Psychol.

[13] Nutbeam D (1986). Health promotion glossary. Health Promot.

[14] Zoffmann V, Kirkevold M (2005). Life versus disease in difficult diabetes care: conflicting perspectives disempower patients and professionals in problem solving. Qual Health Res.

[15] Zoffmann V, Lauritzen T (2006). Guided self-determination improves life skills with Type 1 diabetes and A1C in randomized controlled trial. Patient Educ Couns.

[16] Zoffmann V, Prip A, Christiansen AW (2015). Dramatic change in a young woman’s perception of her diabetes and remarkable reduction in HbA1c after an individual course of guided self-determination. BMJ Case Rep.

[17] Ryan RMD, Edward L. Self-determination theory - basic psychological needs in motivation, development and wellness. the Guilford Press; 2017.

[18] Hrobjartsson A, Emanuelsson F, Skou Thomsen AS, Hilden J, Brorson S (2014). Bias due to lack of patient blinding in clinical trials. A systematic review of trials randomizing patients to blind and nonblind sub-studies. Int J Epidemiol.

[19] Ng JY, Ntoumanis N, Thogersen-Ntoumani C, Deci EL, Ryan RM, Duda JL (2012). Self-determination theory applied to health contexts: a meta-analysis. Perspect Psychol Sci.

[20] Cochrane handbook for systematic reviews of interventions: Cochrane book series. Higgins JPG, S, editor. The Cochrane Collaboration; 2011. Available from www.training.cochrane.org/handbook. Updated March 2011.

[21] Higgins JP, Thompson SG (2002). Quantifying heterogeneity in a meta-analysis. Stat Med.

[22] Mathiesen AS, Rothmann MJ, Zoffmann V, Jakobsen JC, Gluud C, Lindschou J (2021). Self-determination theory interventions versus usual care in people with diabetes: a protocol for a systematic review with meta-analysis and trial sequential analysis. Syst Rev.

[23] Vanroy J, Seghers J, Bogaerts A, Devloo K, De Cock S, Boen F (2017). Short- and long-term effects of a need-supportive physical activity intervention among patients with type 2 diabetes mellitus: a randomized controlled pilot trial. PLoS One.

[24] Glasgow RE, Nutting PA, King DK, Nelson CC, Cutter G, Gaglio B (2005). Randomized effectiveness trial of a computer-assisted intervention to improve diabetes care. Diabetes Care.

[25] Page MJ, McKenzie JE, Bossuyt PM, Boutron I, Hoffmann TC, Mulrow CD (2021). The PRISMA 2020 statement: an updated guideline for reporting systematic reviews. BMJ.

[26] (RevMan). RM. Version 5.3 ed. The Cochrane Collaboration. Copenhagen: The Nordic Cochrane Centre; 2014.

[27] TSA - trial sequential analysis [Web page]. Copenhagen Trial Unit; 2020. Available from: http://ctu.dk/tsa/.

[28] Popp L, Schneider S (2015). Attention placebo control in randomized controlled trials of psychosocial interventions: theory and practice. Trials.

[29] Covidence systematic review software. https://www.covidence.org/. Melbourne: Veritas Health Innovation; 2022.

[30] Brorson AL, Leksell J, AnderssonFranko M, Olinder ALA (2019). person-centered education for adolescents with type 1 diabetes - a randomized controlled trial. Pediatr Diabetes.

[31] Husted GR, Thorsteinsson B, Esbensen BA, Gluud C, Winkel P, Hommel E (2014). Effect of guided self-determination youth intervention integrated into outpatient visits versus treatment as usual on glycemic control and life skills: a randomized clinical trial in adolescents with type 1 diabetes. Trials.

[32] Mohn J, Graue M, Assmus J, Zoffmann V, Thordarson H, Peyrot M (2017). The effect of guided self-determination on self-management in persons with type 1 diabetes mellitus and HbA1c >/=64 mmol/mol: a group-based randomised controlled trial. BMJ Open.

[33] Zoffmann V, Vistisen D, Due-Christensen M (2015). Flexible guided self-determination intervention for younger adults with poorly controlled type 1 diabetes, decreased HbA1c and psychosocial distress in women but not in men: a real-life RCT. Diabet Med.

[34] Juul L, Maindal HT, Zoffmann V, Frydenberg M, Sandbaek A (2014). Effectiveness of a training course for general practice nurses in motivation support in type 2 diabetes care: a cluster-randomised trial. PLoS One.

[35] Mathiesen AS. Vulnerable people with type 2 diabetes: implications and feasibility of a guided self-determination intervention for reducing diabetes distress. Faculty of Public Health, University of Copenhagen; 2019. https://www.researchgate.net/publication/339627249_PhD_Thesis_Title_Vulnerable_people_with_type_2_diabetes_Implications_and_feasibility_of_a_guided_self-_determination_intervention_for_reducing_diabetes_distress.

[36] Nansel TR, Laffel LM, Haynie DL, Mehta SN, Lipsky LM, Volkening LK (2015). Improving dietary quality in youth with type 1 diabetes: randomized clinical trial of a family-based behavioral intervention. Int J Behav Nutr Phys Act.

[37] Yun Q, Ji Y, Liu S, Shen Y, Jiang X, Fan X (2020). Can autonomy support have an effect on type 2 diabetes glycemic control? Results of a cluster randomized controlled trial. BMJ Open Diabetes Res Care.

[38] Elbourne DR, Altman DG, Higgins JP, Curtin F, Worthington HV, Vail A (2002). Meta-analyses involving cross-over trials: methodological issues. Int J Epidemiol.

[39] Schulz KF, Chalmers I, Hayes RJ, Altman DG (1995). Empirical evidence of bias. Dimensions of methodological quality associated with estimates of treatment effects in controlled trials. JAMA.

[40] Moher D, Pham B, Jones A, Cook DJ, Jadad AR, Moher M (1998). Does quality of reports of randomised trials affect estimates of intervention efficacy reported in meta-analyses?. Lancet.

[41] Kjaergard LL, Villumsen J, Gluud C (2001). Reported methodologic quality and discrepancies between large and small randomized trials in meta-analyses. Ann Intern Med.

[42] Gluud LL, Thorlund K, Gluud C, Woods L, Harris R, Sterne JA (2008). Correction: reported methodologic quality and discrepancies between large and small randomized trials in meta-analyses. Ann Intern Med.

[43] Wood L, Egger M, Gluud LL, Schulz KF, Juni P, Altman DG (2008). Empirical evidence of bias in treatment effect estimates in controlled trials with different interventions and outcomes: meta-epidemiological study. BMJ.

[44] Savovic J, Jones H, Altman D, Harris R, Juni P, Pildal J (2012). Influence of reported study design characteristics on intervention effect estimates from randomised controlled trials: combined analysis of meta-epidemiological studies. Health Technol Assess.

[45] Hrobjartsson A, Thomsen AS, Emanuelsson F, Tendal B, Hilden J, Boutron I (2013). Observer bias in randomized clinical trials with measurement scale outcomes: a systematic review of trials with both blinded and nonblinded assessors. CMAJ.

[46] Hrobjartsson A, Thomsen AS, Emanuelsson F, Tendal B, Rasmussen JV, Hilden J (2014). Observer bias in randomized clinical trials with time-to-event outcomes: systematic review of trials with both blinded and non-blinded outcome assessors. Int J Epidemiol.

[47] Savovic J, Turner RM, Mawdsley D, Jones HE, Beynon R, Higgins JPT (2018). Association between risk-of-bias assessments and results of randomized trials in Cochrane reviews: the ROBES meta-epidemiologic study. Am J Epidemiol.

[48] Jakobsen JC, Wetterslev J, Winkel P, Lange T, Gluud C (2014). Thresholds for statistical and clinical significance in systematic reviews with meta-analytic methods. BMC Med Res Methodol.

[49] Higgins JP, Thompson SG, Deeks JJ, Altman DG (2003). Measuring inconsistency in meta-analyses. BMJ.

[50] Keus F, Wetterslev J, Gluud C, van Laarhoven CJ (2010). Evidence at a glance: error matrix approach for overviewing available evidence. BMC Med Res Methodol.

[51] DerSimonian R, Laird N (1986). Meta-analysis in clinical trials. Control Clin Trials.

[52] Demets DL (1987). Methods for combining randomized clinical trials: strengths and limitations. Stat Med.

[53] Imberger G, Thorlund K, Gluud C, Wetterslev J (2016). False-positive findings in Cochrane meta-analyses with and without application of trial sequential analysis: an empirical review. BMJ Open.

[54] Thorlund K, Engstrøm J, Wetterslev J, Brok J, Imberger G, Gluud C. User manual for trial sequential analysis (TSA). 2011. http://www.ctu.dk/tsa/files/tsa_manual.pdf.

[55] Guyatt GH, Oxman AD, Vist GE, Kunz R, Falck-Ytter Y, Alonso-Coello P (2008). GRADE: an emerging consensus on rating quality of evidence and strength of recommendations. BMJ.

[56] GRADEpro [Software]. McMaster University; 2015 (developed by Evidence Prime, Inc.) Available from: https://www.gradepro.org.

[57] Moher D, Shamseer L, Clarke M, Ghersi D, Liberati A, Petticrew M (2015). Preferred reporting items for systematic review and meta-analysis protocols (PRISMA-P) 2015 statement. Syst Rev.

[58] Husted GR, Thorsteinsson B, Esbensen BA, Hommel E, Zoffmann V (2011). Improving glycaemic control and life skills in adolescents with type 1 diabetes: a randomised, controlled intervention study using the guided self-determination-young method in triads of adolescents, parents and health care providers integrated into routine paediatric outpatient clinics. BMC Pediatr.

[59] Mathiesen AS, Egerod I, Jensen T, Kaldan G, Langberg H, Thomsen T (2019). Psychosocial interventions for reducing diabetes distress in vulnerable people with type 2 diabetes mellitus: a systematic review and meta-analysis. Diabetes Metab Syndr Obes.

[60] Mathiesen AS, Zoffmann V, Skytte TB, Jakobsen JC, Gluud C, Lindschou J (2021). Guided self-determination intervention versus attention control for people with type 2 diabetes in outpatient clinics: a protocol for a randomised clinical trial. BMJ Open.

[61] Nano J, Carinci F, Okunade O, Whittaker S, Walbaum M, Barnard-Kelly K (2020). A standard set of person-centred outcomes for diabetes mellitus: results of an international and unified approach. Diabet Med.

[62] Dennick K, Bridle C, Sturt J (2015). Written emotional disclosure for adults with type 2 diabetes: a primary care feasibility study. Prim Health Care Res Dev.

[63] Fisher L, Polonsky WH, Hessler D (2019). Addressing diabetes distress in clinical care: a practical guide. Diabet Med.

[64] Due-Christensen M, Zoffmann V, Hommel E, Lau M (2012). Can sharing experiences in groups reduce the burden of living with diabetes, regardless of glycaemic control?. Diabet Med.

[65] Fisher EB, Thorpe CT, Devellis BM, Devellis RF (2007). Healthy coping, negative emotions, and diabetes management: a systematic review and appraisal. The Diabetes Educ.

[66] McSharry J, Dinneen SF, Humphreys M, O’Donnell M, O’Hara MC, Smith SM (2019). Barriers and facilitators to attendance at type 2 diabetes structured education programmes: a qualitative study of educators and attendees. Diabet Med.

[67] Kirsh SR, Aron DC, Johnson KD, Santurri LE, Stevenson LD, Jones KR (2017). A realist review of shared medical appointments: how, for whom, and under what circumstances do they work?. BMC Health Serv Res.

