# Supplementary material for: Self-determination theory interventions versus usual care in people with diabetes: a systematic review with meta-analysis and trial sequential analysis
Source: Syst Rev. 2023 Sep 6;12:158. doi: 10.1186/s13643-023-02308-z (PMC10483731; doi:10.1186/s13643-023-02308-z)
Supplement: Supplementary file 1 — Additional file 1. Search strategies. [file 13643_2023_2308_MOESM1_ESM.doc]

# Search strategies for

# Self-determination theory interventions versus usual care in adults with diabetes

# (Anne Sophie Mathiesen)

# Preliminary searches performed April 2022

**Total number of references identified: 5463 references**

**Number of duplicates excluded: 1587 references**

**Number of references in final list: 3876 references**

Cochrane Central Register of Controlled Trials (CENTRAL; 2022, Issue 4) in the Cochrane Library (2106 hits)

#1 MeSH descriptor: [Diabetes Mellitus] explode all trees

#2 (diabet* or IDDM or NIDDM or MODY or T1D* or T2D* or insulin* depend* or insulin?depend*)

#3 #1 or #2

#4 MeSH descriptor: [Motivation] explode all trees

#5 MeSH descriptor: [Personal Autonomy] explode all trees

#6 MeSH descriptor: [Self Care] explode all trees

#7 MeSH descriptor: [Empowerment] explode all trees

#8 (self?determination* or empowerment* or life?skill* or consultation near home or reflection?sheet*)

#9 #4 or #5 or #6 or #7 or #8

#10 #3 and #9

#11 (child* or p*ediat* or neonat* or newborn* or infant*) not (adolesc* or teen* or adult* or aged or elder* or middle next age* or old next age)

#12 #10 not #11

**MEDLINE Ovid (1946 to April 2022) (401 hits)**

1. exp Diabetes Mellitus/

2. (diabet* or IDDM or NIDDM or MODY or T1D* or T2D* or insulin* depend* or insulin?depend*).mp. [mp=title, abstract, original title, name of substance word, subject heading word, floating sub-heading word, keyword heading word, organism supplementary concept word, protocol supplementary concept word, rare disease supplementary concept word, unique identifier, synonyms]

3. 1 or 2

4. exp Motivation/px [Psychology]

5. exp personal autonomy/

6. exp Self Care/px [Psychology]

7. exp Empowerment/

8. (self?determination* or empowerment* or life?skill* or consultation near home or reflection?sheet*).mp. [mp=title, abstract, original title, name of substance word, subject heading word, floating sub-heading word, keyword heading word, organism supplementary concept word, protocol supplementary concept word, rare disease supplementary concept word, unique identifier, synonyms]

9. 4 or 5 or 6 or 7 or 8

10. 3 and 9

11. limit 10 to (humans and ("adolescent (13 to 18 years)" or "young adult (19 to 24 years)" or "adult (19 to 44 years)" or "young adult and adult (19-24 and 19-44)" or "middle age (45 to 64 years)" or "middle aged (45 plus years)" or "all aged (65 and over)" or "aged (80 and over)"))

12. (randomized controlled trial or controlled clinical trial).pt. or clinical trials as topic.sh. or trial.ti.

13. (random* or blind* or placebo* or meta-analys*).mp. [mp=title, abstract, original title, name of substance word, subject heading word, floating sub-heading word, keyword heading word, organism supplementary concept word, protocol supplementary concept word, rare disease supplementary concept word, unique identifier, synonyms]

14. 11 and (12 or 13)

**Embase Ovid (1974 to April 2022) (1792 hits)**

1. exp diabetes mellitus/

2. (diabet* or IDDM or NIDDM or MODY or T1D* or T2D* or insulin* depend* or insulin?depend*).mp. [mp=title, abstract, heading word, drug trade name, original title, device manufacturer, drug manufacturer, device trade name, keyword, floating subheading word, candidate term word]

3. 1 or 2

4. exp motivation/

5. exp personal autonomy/

6. exp self care/

7. exp empowerment/

8. (self?determination* or empowerment* or life?skill* or consultation near home or reflection?sheet*).mp. [mp=title, abstract, heading word, drug trade name, original title, device manufacturer, drug manufacturer, device trade name, keyword, floating subheading word, candidate term word]

9. 4 or 5 or 6 or 7 or 8

10. 3 and 9

11. limit 10 to (human and (adult <18 to 64 years> or aged <65+ years>))

12. Randomized controlled trial/ or Controlled clinical study/ or trial.ti.

13. (random* or blind* or placebo* or meta-analys*).mp. [mp=title, abstract, heading word, drug trade name, original title, device manufacturer, drug manufacturer, device trade name, keyword, floating subheading word, candidate term word]

14. 11 and (12 or 13)

**LILACS (Bireme; 1982 to April 2022) (47 hits)**

(diabet$ or IDDM or NIDDM or MODY or T1D$ or T2D$ or insulin$ depend$ or insulin$depend$) [Words] and (self$determination$ or empowerment$ or life$skill$ or consultation near home or reflection$sheet$) [Words]

Science Citation Index Expanded (1900 to June 2020) and Conference Proceedings Citation Index – Science (1990 to April 2022) (Web of Science) (247 hits)

#7 #6 AND #5

#6 TI=(random* or blind* or placebo* or meta-analys* or trial*) OR TS=(random* or blind* or placebo* or meta-analys*)

#5 #3 NOT #4

#4 TS=((child* or pediat* or paediat* or neonat* or newborn* or infant*) not (adolesc* or teen* or adult* or aged or elder* or "middle age*" or "old age"))

#3 #2 AND #1

#2 TS=(self$determination* or empowerment* or life$skill* or consultation near home or reflection$sheet*)

#1 TS=(diabet* or IDDM or NIDDM or MODY or T1D* or T2D* or insulin* depend* or insulin$depend*)

PsycInfo (link to searches) (344 hits)

https://search.ebscohost.com/login.aspx?direct=true&db=psyh&bquery=(((DE+%26quot%3bDiabetes+Mellitus%26quot%3b+OR+DE+%26quot%3bGestational+Diabetes%26quot%3b+OR+DE+%26quot%3bType+2+Diabetes%26quot%3b)+OR+((diabet*)+OR+(iddm)+OR+(niddm)+OR+(mody)+OR+(t1d*)+OR+(t2D*)+OR+(insulin*)+OR+(depend*)+OR+(insulin+depend*)))+AND+((DE+%26quot%3bGoal+Setting%26quot%3b+OR+DE+%26quot%3bAchievement+Motivation%26quot%3b+OR+DE+%26quot%3bExtrinsic+Motivation%26quot%3b+OR+DE+%26quot%3bGoals%26quot%3b+OR+DE+%26quot%3bIntrinsic+Motivation%26quot%3b+OR+DE+%26quot%3bPsychological+Needs%26quot%3b+OR+DE+%26quot%3bMotivation%26quot%3b+OR+DE+%26quot%3bMotivation+Training%26quot%3b+OR+DE+%26quot%3bMotivational+Interviewing%26quot%3b)+OR+(DE+%26quot%3bAutonomy%26quot%3b+OR+DE+%26quot%3bEmpowerment%26quot%3b+OR+DE+%26quot%3bIndependence+(Personality)%26quot%3b)+OR+(personal+autonom*)+OR+(DE+%26quot%3bBehavior+Modification%26quot%3b+OR+DE+%26quot%3bSelf-Determination%26quot%3b+OR+DE+%26quot%3bSelf-Management%26quot%3b+OR+DE+%26quot%3bSelf-Monitoring%26quot%3b+OR+DE+%26quot%3bSelf-Regulation%26quot%3b)+OR+(reflection+sheets)))+AND+(((randomized+controlled+trial)+OR+(controlled+clinical+trial))+OR+(DE+%26quot%3bRandomized+Controlled+Trials%26quot%3b+OR+DE+%26quot%3bClinical+Trials%26quot%3b+OR+DE+%26quot%3bRandomized+Clinical+Trials%26quot%3b))&type=1&searchMode=Standard&site=ehost-live&ssl=y>S9 AND (S10 OR S11)</A>

CINAHL (link to searches) (526 hits)

http://search.ebscohost.com/login.aspx?direct=true&db=rzh&bquery=((MH+%26quot%3bRandomized+Controlled+Trials%2b%26quot%3b)+OR+(MH+%26quot%3bClinical+Trials%2b%26quot%3b))+AND+((((MH+%26quot%3bDiabetes+Mellitus%2c+Type+1%26quot%3b)+OR+(MH+%26quot%3bDiabetes+Mellitus%2c+Type+2%26quot%3b)+OR+(MH+%26quot%3bDiabetes+Mellitus%26quot%3b))+OR+((diabet*)+OR+(IDDM)+OR+(NIDDM)+OR+(MODY)+OR+(T1D*)+OR+(T2D*)+OR+(Insulin*+depend*)))+AND+(((MH+%26quot%3bMotivation%26quot%3b))+OR+((MH+%26quot%3bMotivational+Interviewing%26quot%3b))+OR+((MH+%26quot%3bPatient+Autonomy%26quot%3b))+OR+(personal+autonom*)+OR+((MH+%26quot%3bBlood+Glucose+Self-Monitoring%26quot%3b)+OR+(MH+%26quot%3bSelf-Management%26quot%3b))+OR+self-determination+OR+empowerment*+OR+(life+skills)+OR+lifeskills+OR+(reflection+sheets)+OR+((MH+%26quot%3bEmpowerment%26quot%3b))))&type=1&searchMode=Standard&site=ehost-live
