# Supplementary material for: Self-determination theory interventions versus usual care in people with diabetes: a systematic review with meta-analysis and trial sequential analysis
Source: Syst Rev. 2023 Sep 6;12:158. doi: 10.1186/s13643-023-02308-z (PMC10483731; doi:10.1186/s13643-023-02308-z)
Supplement: Supplementary file 3 — Additional file 3. Sensitivity analyses best-worst and worst-best scenarios Quality of life and diabetes distress. [file 13643_2023_2308_MOESM3_ESM.docx]

**Supplementary file 3**

**Sensitivity analyses best-worst and worst-best scenarios Quality of life and diabetes distress**

**Quality of life, end of intervention (experimental (best), control (worst))**

**
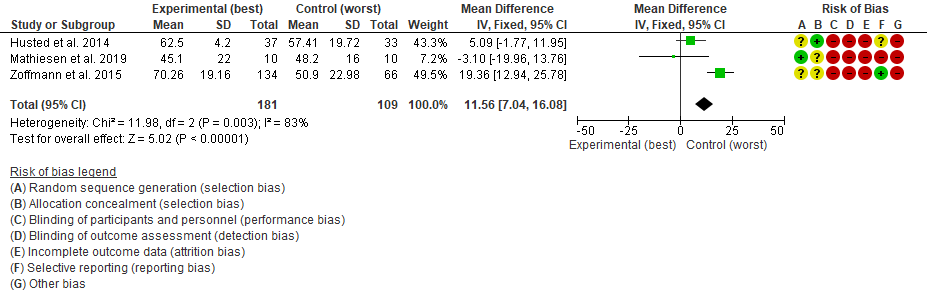
**

**Quality of life, end of intervention (experimental (worst), control (best))**

**
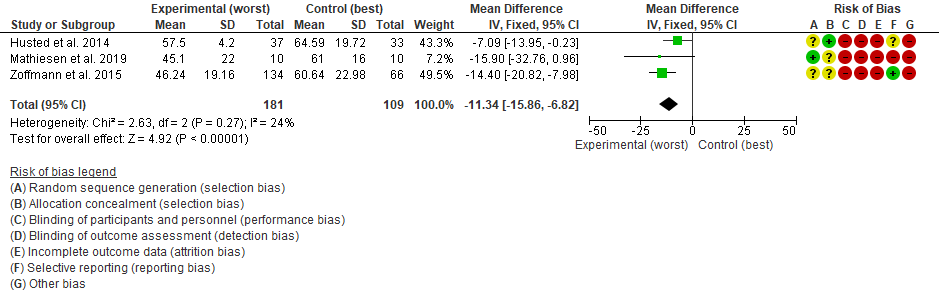
**

**Quality of life, longest follow-up (experimental (best), control (worst))**

**
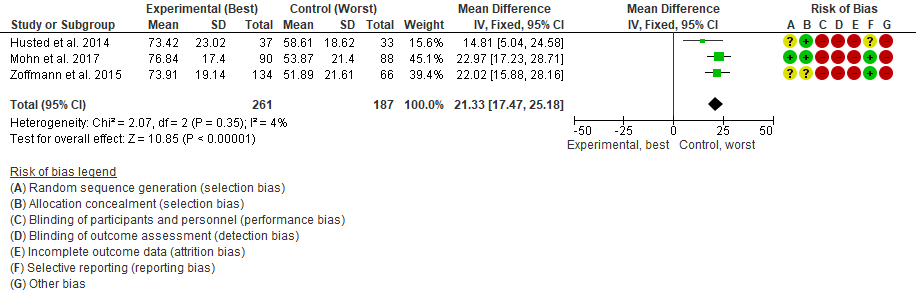
**

**Quality of life, longest follow-up (experimental (worst), control (best))**

**
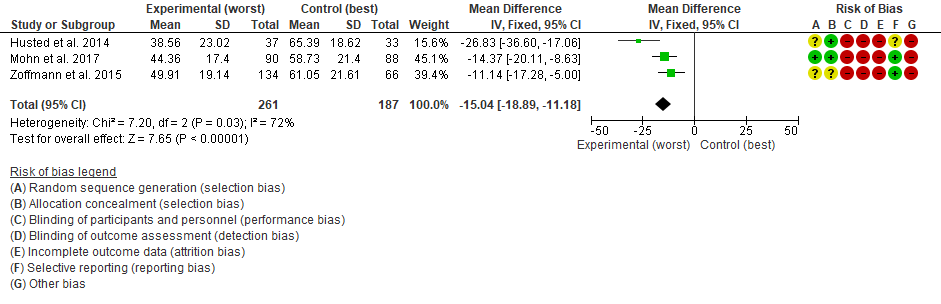
**

**Diabetes distress, end of intervention (experimental (best), control (worst))**

**
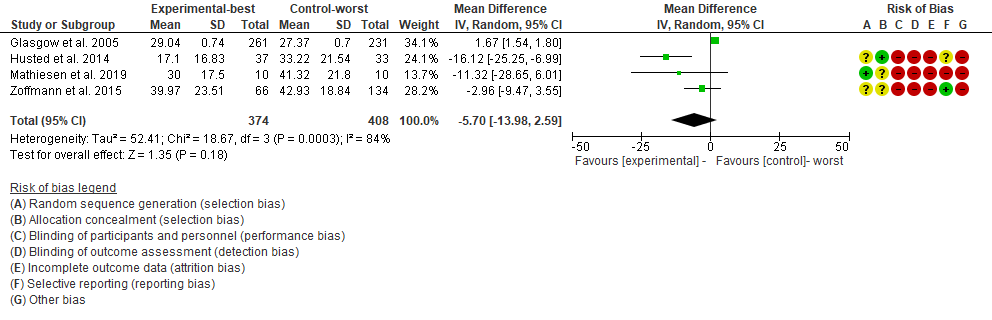
**

**Diabetes distress, end of intervention (experimental (worst), control (best))**

**
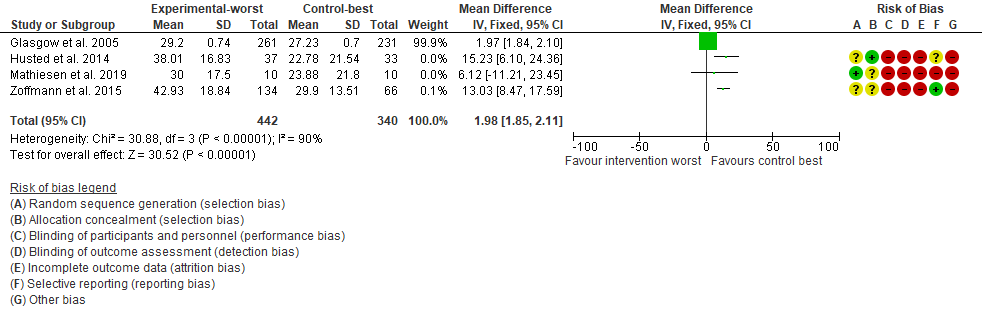
**

**Diabetes distress, longest follow-up (experimental (best), control (worst))**

**
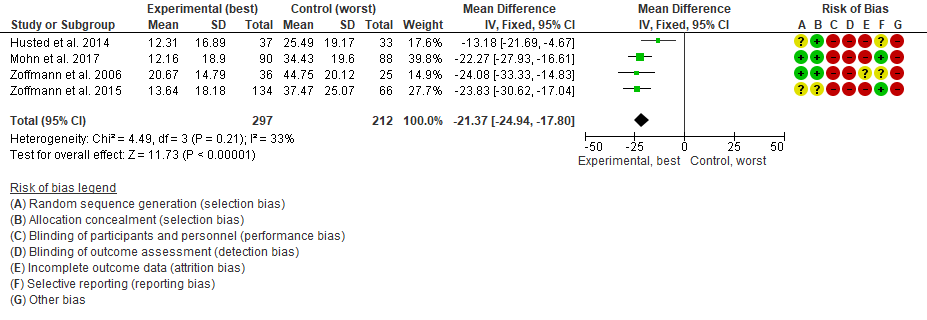
**

**Diabetes distress, longest follow-up (experimental (worst), control (best))**

**
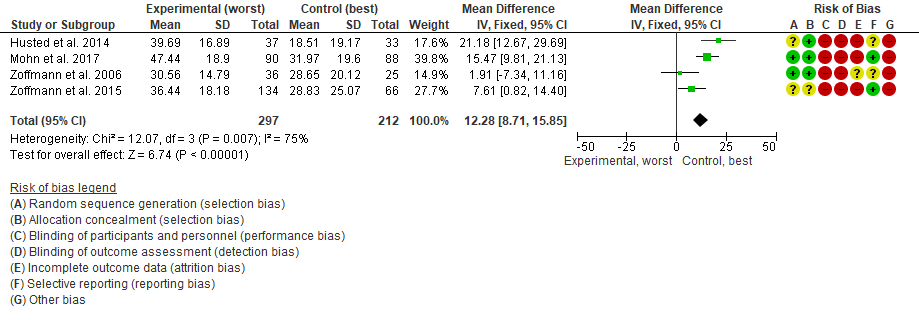
**
