# Supplementary material for: Self-determination theory interventions versus usual care in people with diabetes: a systematic review with meta-analysis and trial sequential analysis
Source: Syst Rev. 2023 Sep 6;12:158. doi: 10.1186/s13643-023-02308-z (PMC10483731; doi:10.1186/s13643-023-02308-z)
Supplement: Supplementary file 4 — Additional file 4. Results all outcomes. [file 13643_2023_2308_MOESM4_ESM.docx]

**Supplementary file 4: Results all outcomes**

**Primary outcomes:**

Figure 2: Meta-analysis and Trial Sequential Analysis of quality of life, end of intervention

**a**

**
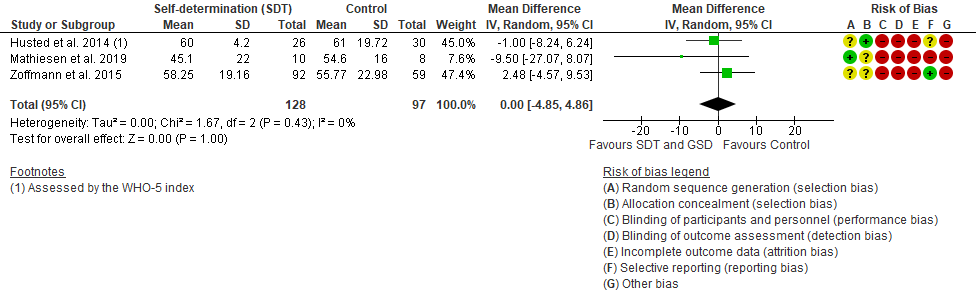
b**


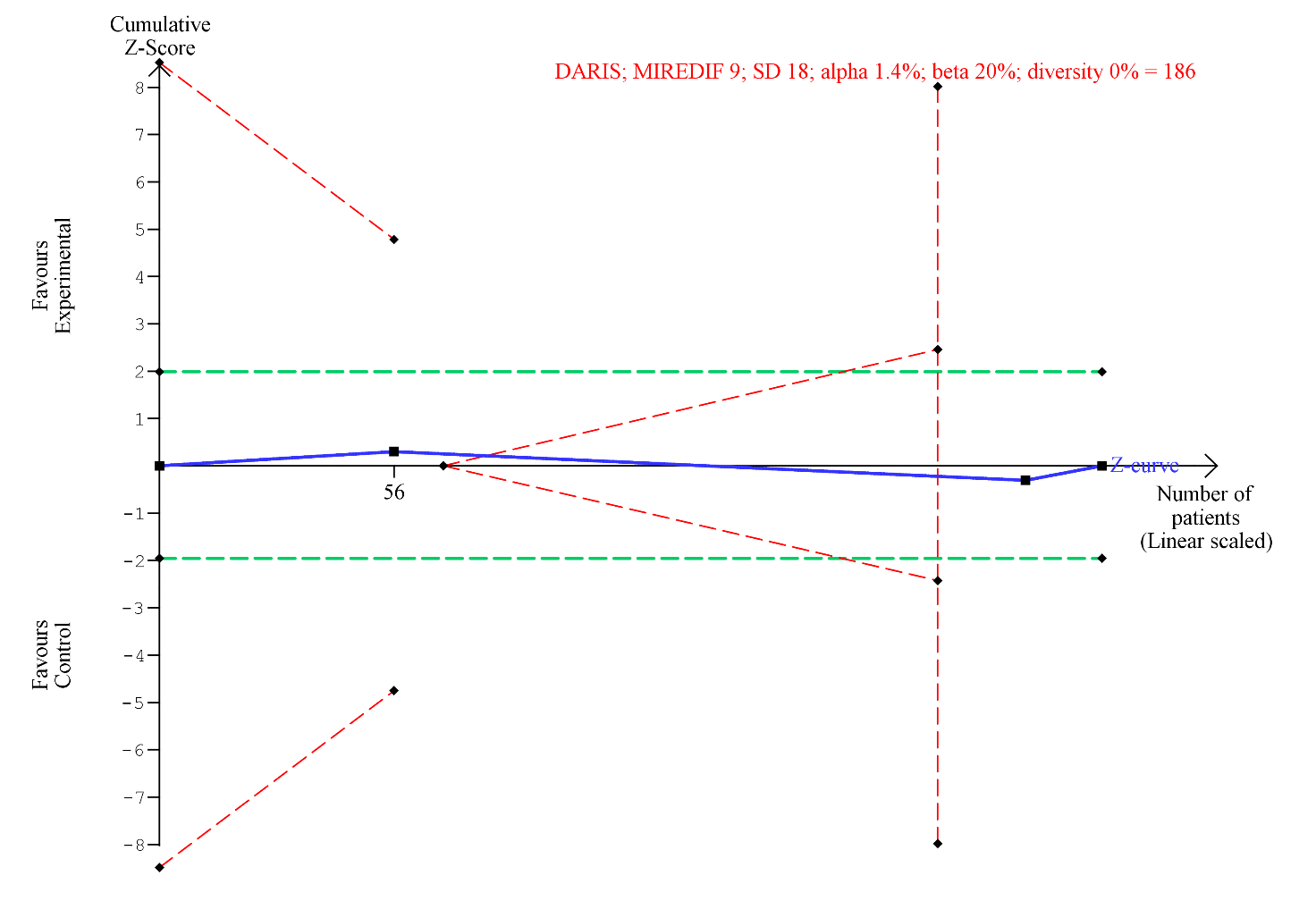


*Figure 2 Meta-analysis and Trial Sequential Analysis (TSA) for quality of life, end of intervention for self-determination theory vs. control.* ***a*** *Meta-analysis.* ***b*** *TSA The diversity- adjusted required information size (DARIS) was calculated according to a mean difference of 9 points, which is half of the observed SD of 18 points, alpha of 1.4%; a beta of 20% (80% power); and diversity 0%. The DARIS was 186 participants. The cumulative Z-curve (blue line) breaches the boundary of futility (dotted outward sloping red lines) and the DARIS. The green dotted lines show naive conventional boundaries (alpha 5%)*

Figure 3. Meta-analysis and Trail Sequential Analysis of quality of life, longest follow-up

**a**

**
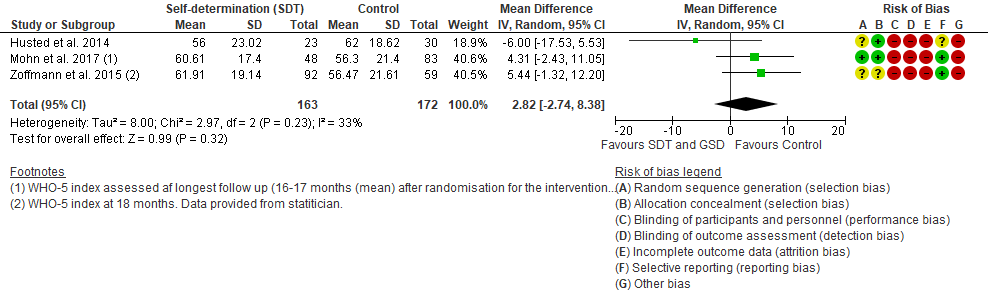
b**

**
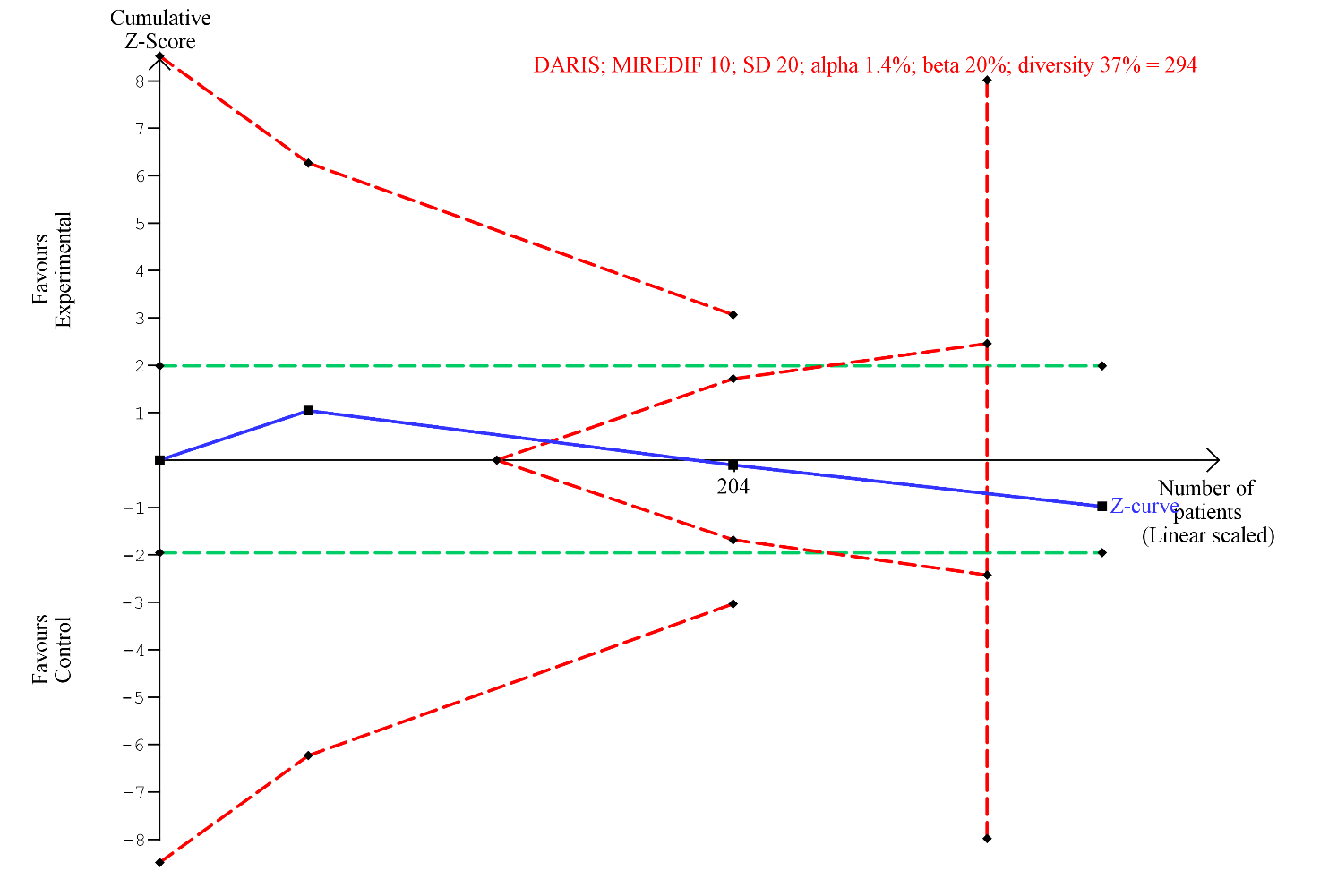
** *Figure 3. Meta-analysis and Trial Sequential Analysis (TSA) for quality of life, longest follow-up for self-determination theory vs. control.* ***a*** *Meta-analysis.* ***b*** *TSA* *The diversity adjusted required information size (DARIS) was calculated according to a mean difference of 10 points, which is half of the observed SD of 20 points, alpha of 1.4%; a beta of 20% (80% power); and diversity 37%. The DARIS was 294 participants. The cumulative Z-curve (blue line) breaches the boundary of futility (dotted outward sloping red lines) and the DARIS. The green dotted lines show naive conventional boundaries (alpha 5%)*

Figure 4. Meta-analysis of all-cause mortality

**a**

**
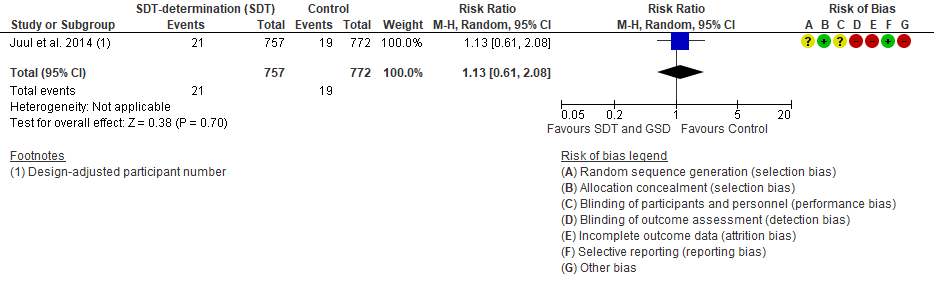
**

*Figure 4. Meta-analysis for all-cause mortality for self-determination theory vs. control.* Trial Sequential Analysis (TSA) not shown due to too little information.

**Serious adverse events**

Reported narratively.

**Secondary outcomes**

Figure 5. Meta-analysis of diabetes distress, end of intervention

**a**


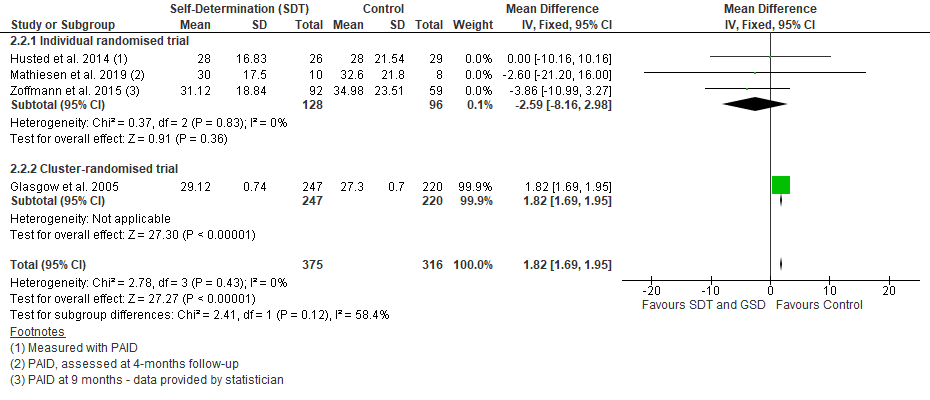


*Figure 5. Meta-analysis for diabetes distress, end of intervention for self-determination theory vs. control.* Trial Sequential Analysis figure not shown as number of included participants exceeded 100% of DARIS

Figure 6. Meta-analysis and Trial Sequential Analysis of diabetes distress, longest follow-up

**a**


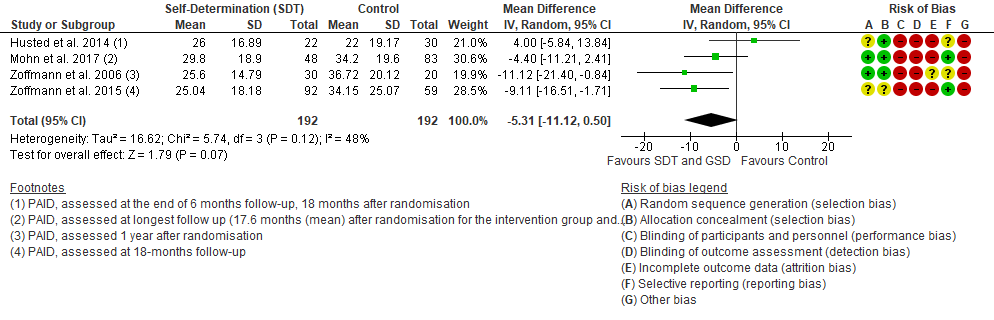


**b**


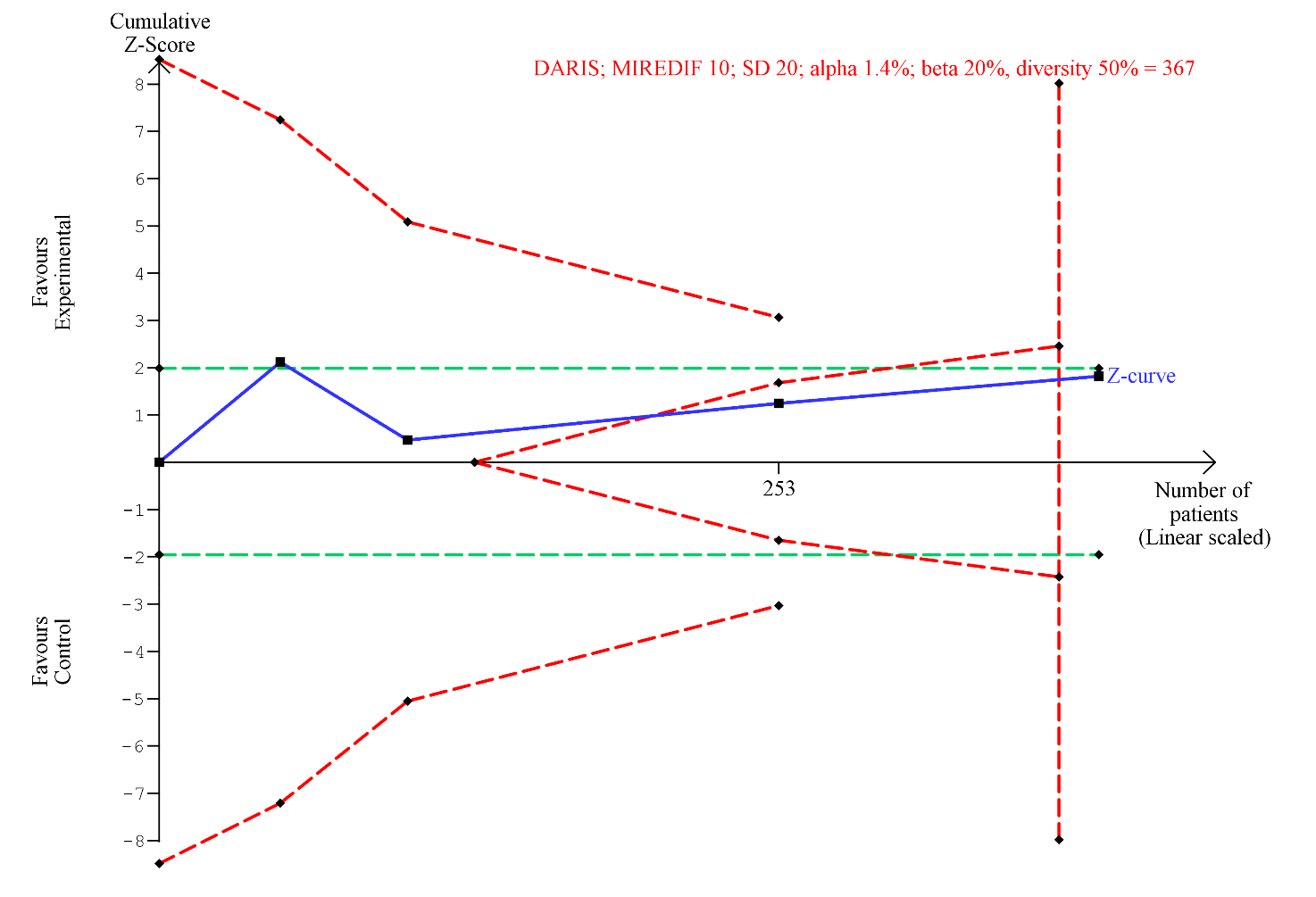


*Figure 6. Meta-analysis and Trial Sequential Analysis (TSA) for diabetes distress, longest follow-up for self-determination theory vs. control.* ***a*** *Meta-analysis.* ***b*** *TSA The diversity adjusted required information size (DARIS) was calculated according to a mean difference of 10 points, which is half of the observed SD of 20 points, alpha of 1.4%; a beta of 20% (80% power); and diversity 50%. The DARIS was 367 participants. The cumulative Z-curve (blue line) breaches the boundary of futility (dotted outward sloping red lines) and the DARIS. The green dotted lines show naive conventional boundaries (alpha 5%)*

**Depressive symptoms at end of intervention and at longest follow-up**

Two trials [1, 2] assessed depressive symptoms at the end of intervention. Glasgow et al. [2] reported depressive symptoms measured by the Patient Health Questionnaire (PHQ-9) but in percent and the authors did not reply to our request for additional data. Mathiesen et al. (2019) measured depressive symptoms by the hospital anxiety and depression score (HADS) at the end of intervention and reported no difference between the intervention and the control group (MD -0.10 points, 95% CI -6.17, 5.97, *p*=0.97) in a high risk of bias small feasibility trial (n=20) [1] .This outcome result was over-all assessed as a high risk of bias and the certainty of the evidence was considered as very low due to very serious risk of bias, serious inconsistency, and serious imprecision (Table 2, Summary of findings). No trials reported on depressive symptoms at longest follow-up.

**Adverse events, not considered serious at end of intervention and at longest follow-up**

Reported narratively.

**Exploratory outcomes**

Figure 7. Meta-analysis and Trial Sequential Analysis of HbA1c, end of intervention.

**a
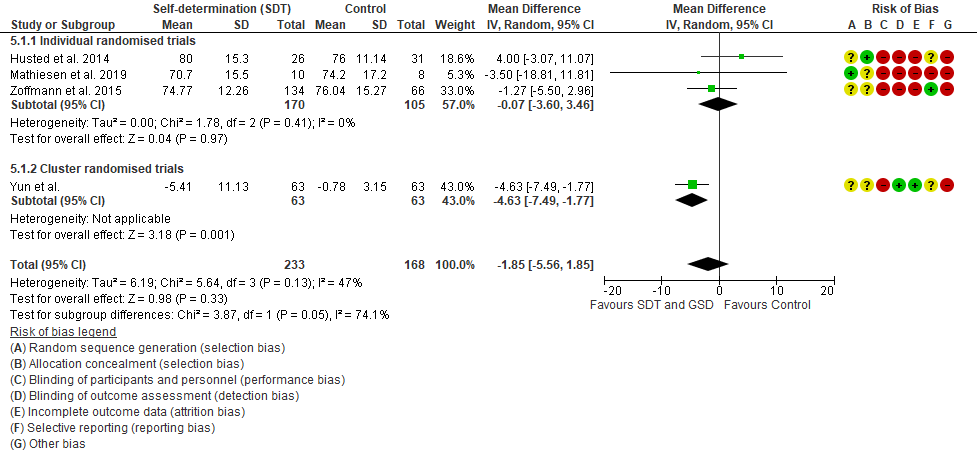
b**
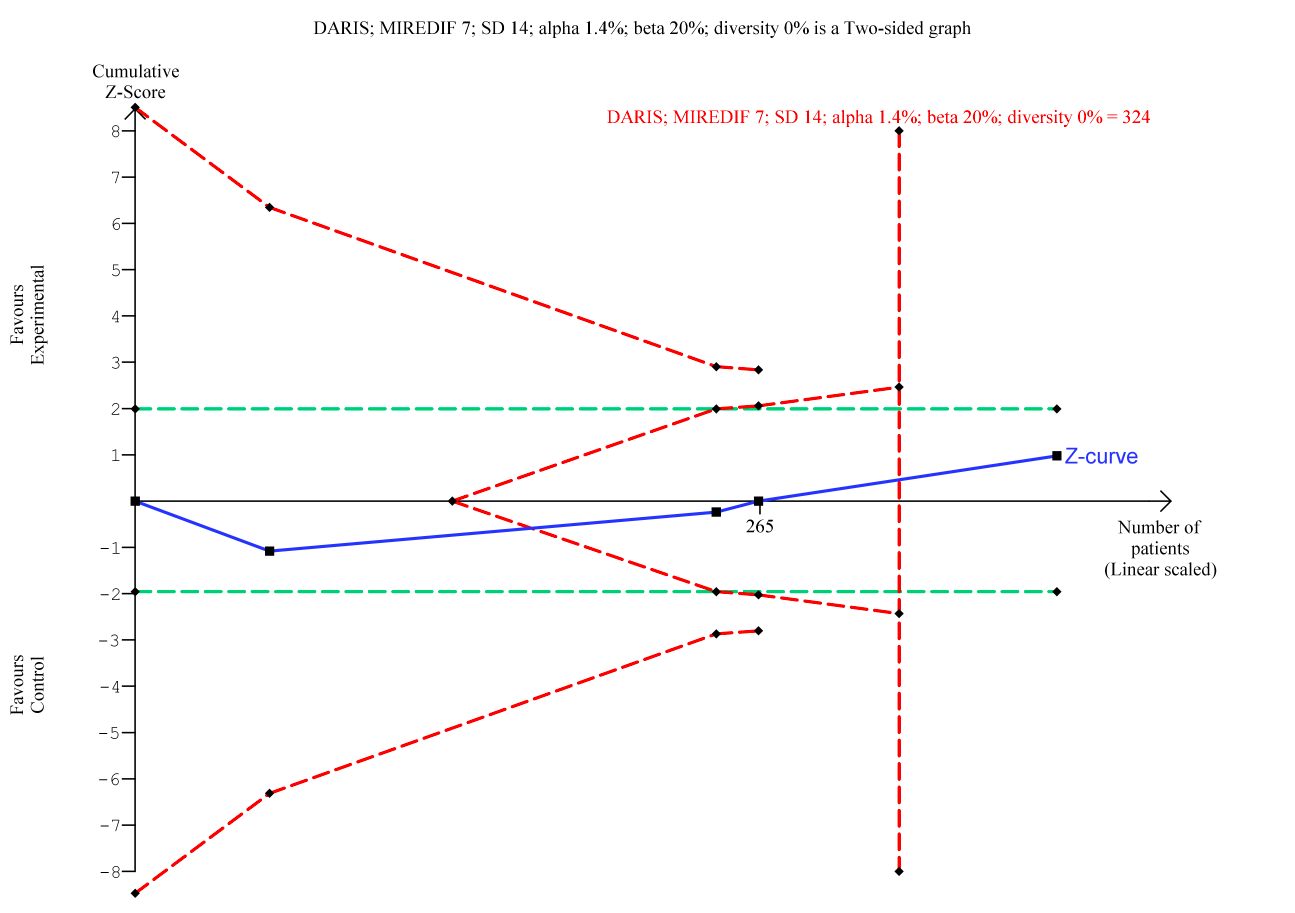


*Figure 7. Meta-analysis and Trial Sequential Analysis (TSA) for HbA1c, end of intervention for self-determination theory vs. control.* ***a*** *Meta-analysis.* ***b*** *TSA The diversity adjusted required information size (DARIS) was calculated according to a mean difference of 7 mmol/mol, which is half of the observed SD of 14 mmol/mol, alpha of 1.4%; a beta of 20% (80% power); and diversity 0%. The DARIS was 324 participants. The cumulative Z-curve (blue line) breaches the boundary of futility (dotted outward sloping red lines) and the DARIS. The green dotted lines show naive conventional boundaries (alpha 5%)*

Figure 8. Meta-analysis and Trial Sequential Analysis of HbA1c, longest follow-up.

**a**


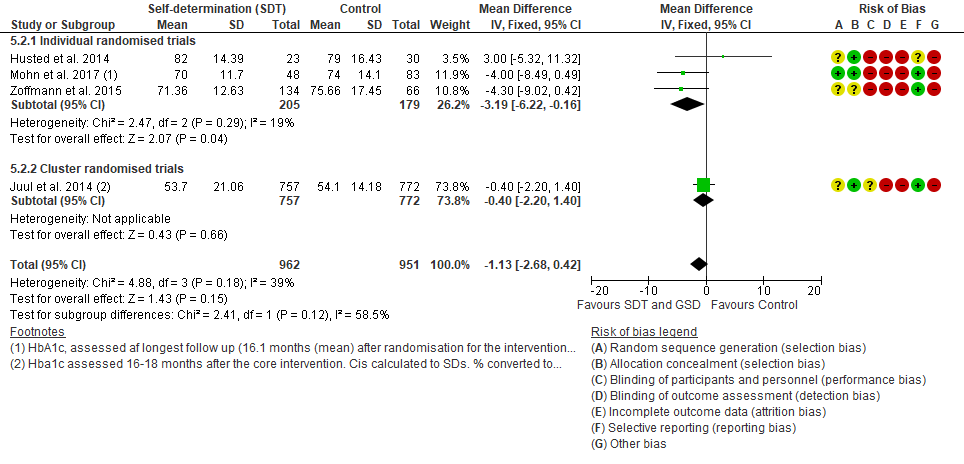


**b**


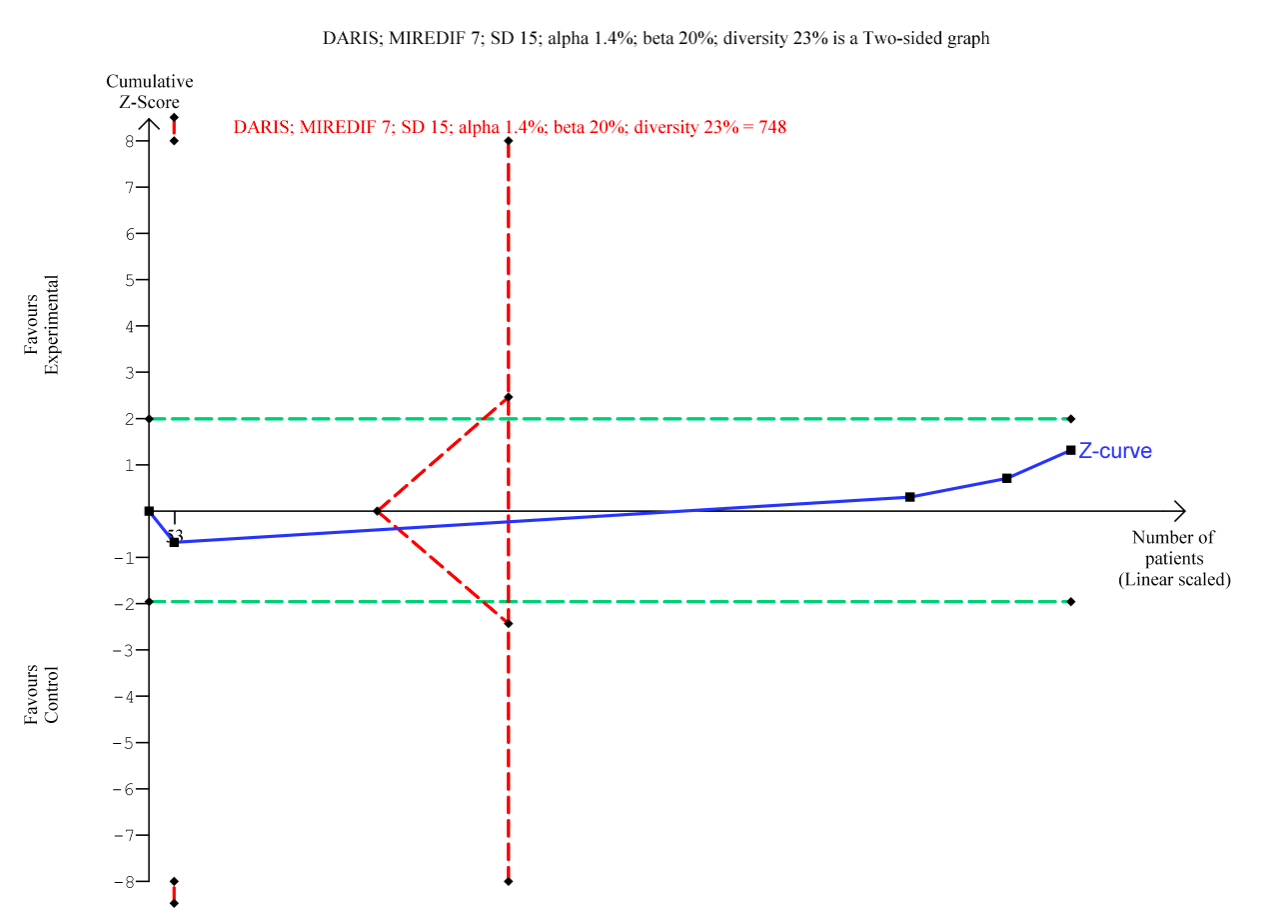


*Figure 8. Meta-analysis and Trial Sequential Analysis (TSA) for HbA1c, longest follow-up for self-determination theory vs. control.* ***a*** *Meta-analysis.* ***b*** *TSA The diversity adjusted required information size (DARIS) was calculated according to a mean difference of 7 mmol/mol, which is half of the observed SD of 15 mmol/mol, alpha of 1.4%; a beta of 20% (80% power); and diversity 23%. The DARIS was 748 participants. The cumulative Z-curve (blue line) breaches the boundary of futility (dotted outward sloping red lines) and the DARIS. The green dotted lines show naive conventional boundaries (alpha 5%)*

Figure 9. Meta-analysis and Trial Sequential Analysis of motivation, autonomy, end of intervention

**a**


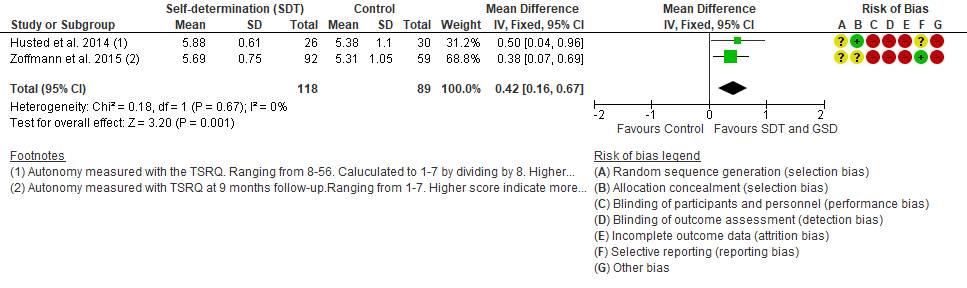


**b**


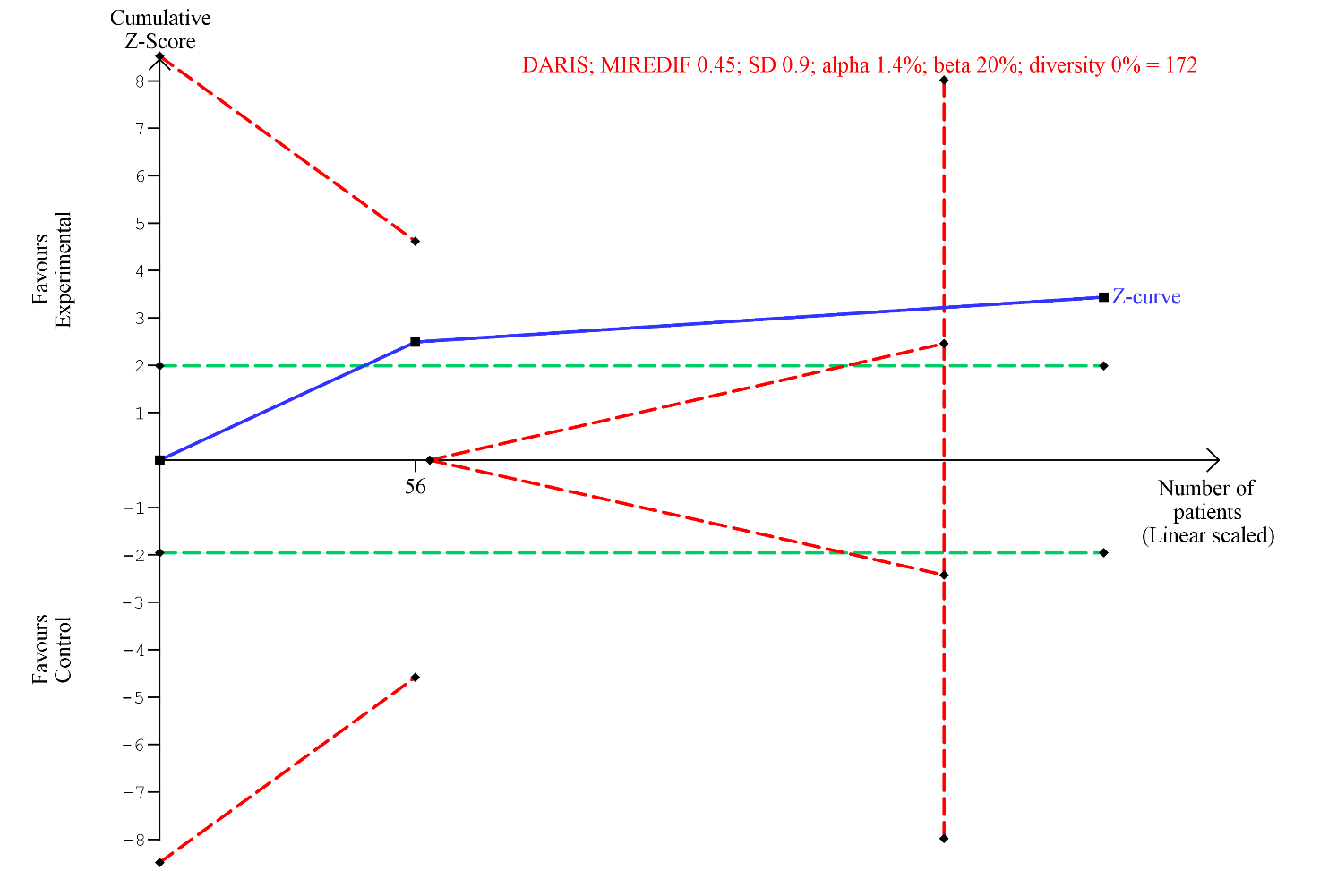


*Figure 9. Meta-analysis and Trial Sequential Analysis (TSA) for motivation (autonomy), end of intervention for self-determination theory vs. control.* ***A*** *Meta-analysis.* ***B*** *TSA The diversity adjusted required information size (DARIS) was calculated according to a mean difference of 0.45 points, which is half of the observed SD 0.9 points, alpha of 1.4%; a beta of 20% (80% power); and diversity 0%. The DARIS was 172 participants. The cumulative Z-curve (blue line) breaches the DARIS for benefit. The green dotted lines show naive conventional boundaries (alpha 5%)*

Figure 10. Meta-analysis and Trial Sequential Analysis of motivation (autonomy), longest follow-up

**a**

**
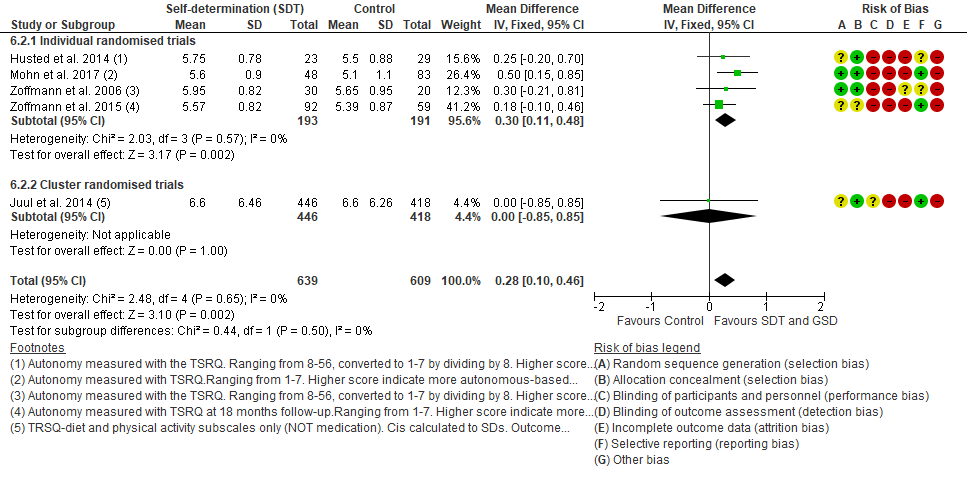
**

**b**


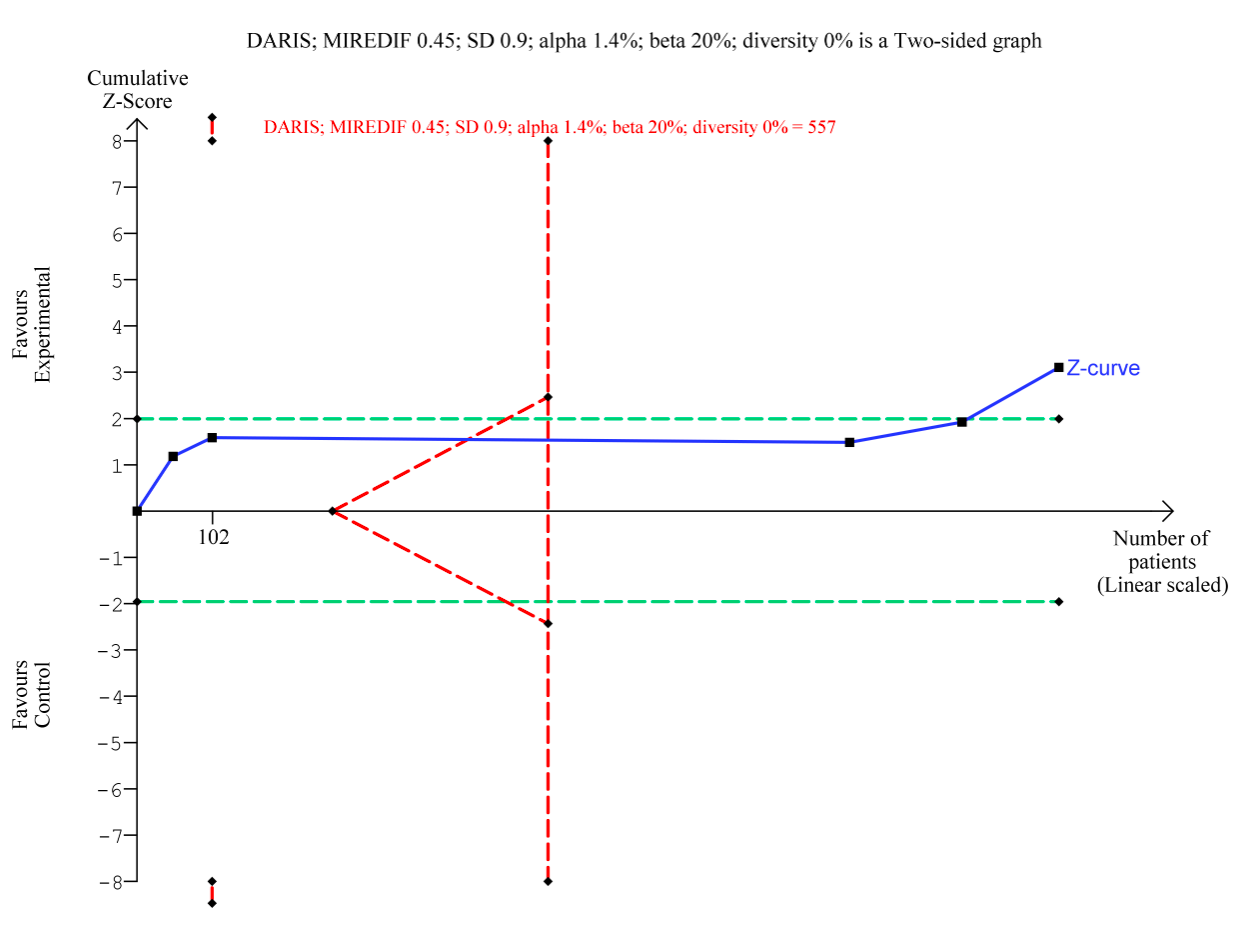


*Figure 10. Meta-analysis and Trial Sequential Analysis (TSA) for motivation (autonomy), longest follow-up for self-determination theory vs. control.* ***A*** *Meta-analysis.* ***B*** *TSA The diversity adjusted required information size (DARIS) was calculated according to a mean difference of 0.45 points, which is half of the observed SD 0.9 points, alpha of 1.4%; a beta of 20% (80% power); and diversity 0%. The DARIS was 557 participants. The cumulative Z-curve (blue line) breaches the DARIS for benefit. The green dotted lines show naive conventional boundaries (alpha 5%)*

Figure 11. Meta-analysis and Trial Sequential Analysis of motivation (control), end of intervention

**a**

**
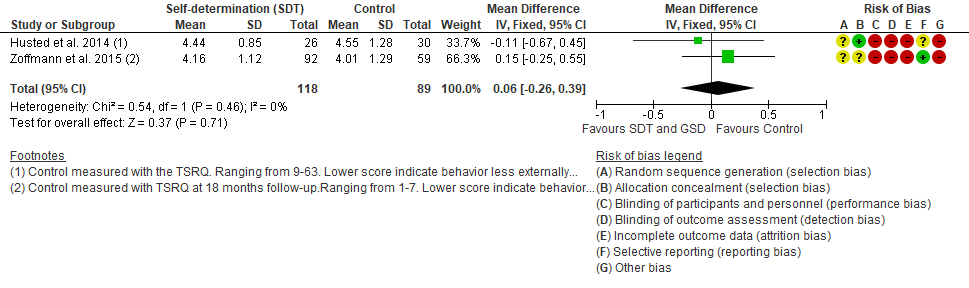
**

**b**


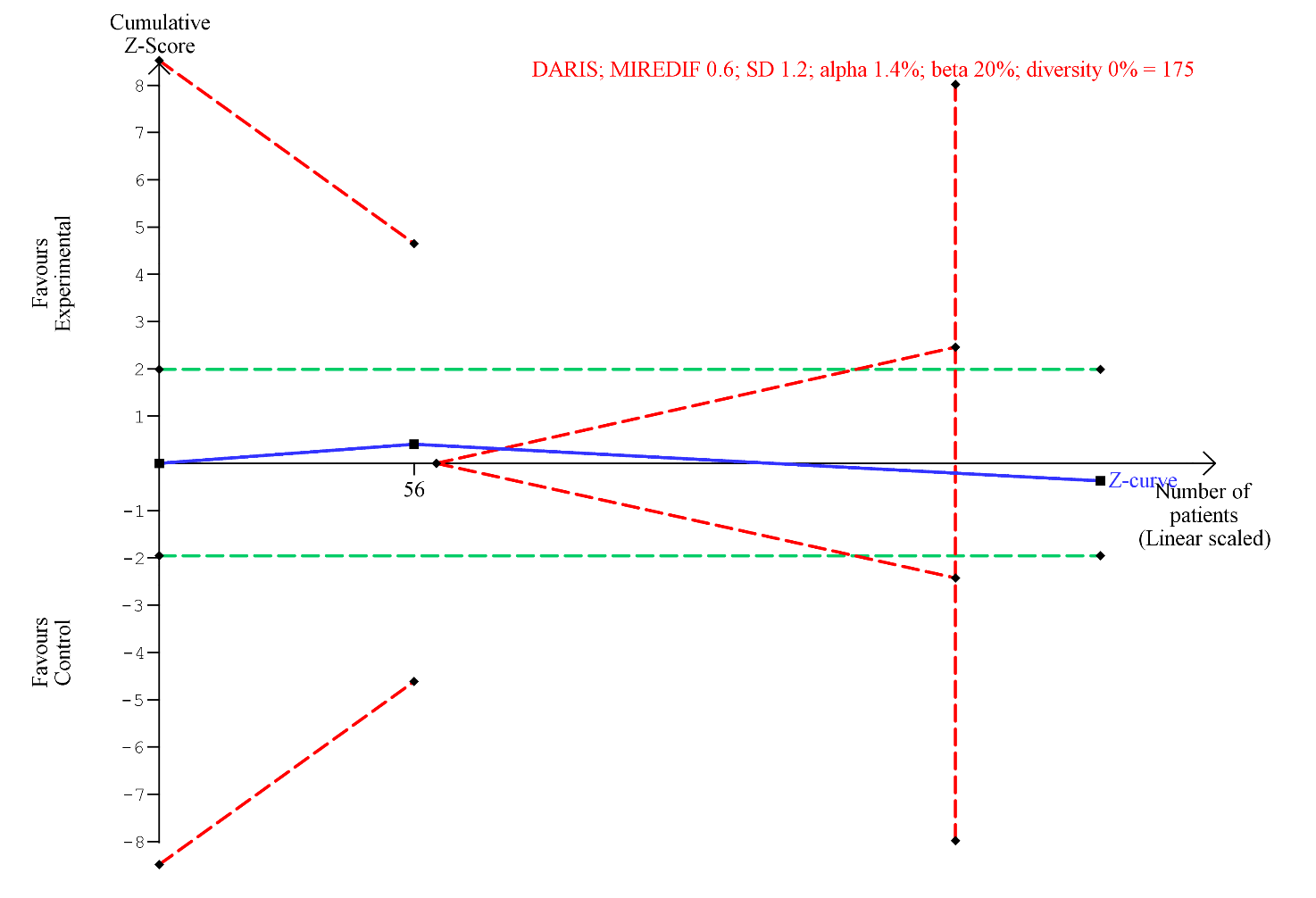
 *Figure 11. Meta-analysis and Trial Sequential Analysis (TSA) for motivation (control), end of intervention for self-determination theory vs. control.* ***a*** *Meta-analysis.* ***b*** *TSA The diversity adjusted required information size (DARIS) was calculated according to a mean difference of 0.6 points, which is half of the observed SD 1.2 points, alpha of 1.4%; a beta of 20% (80% power); and diversity 0%. The DARIS was 175 participants. The cumulative Z-curve (blue line) breaches the boundary of futility (dotted outward sloping red lines) and the DARIS. The green dotted lines show naive conventional boundaries (alpha 5%)*

Figure 12. Meta-analysis and Trial Sequential Analysis of motivation (control), longest follow-up

**a**

**
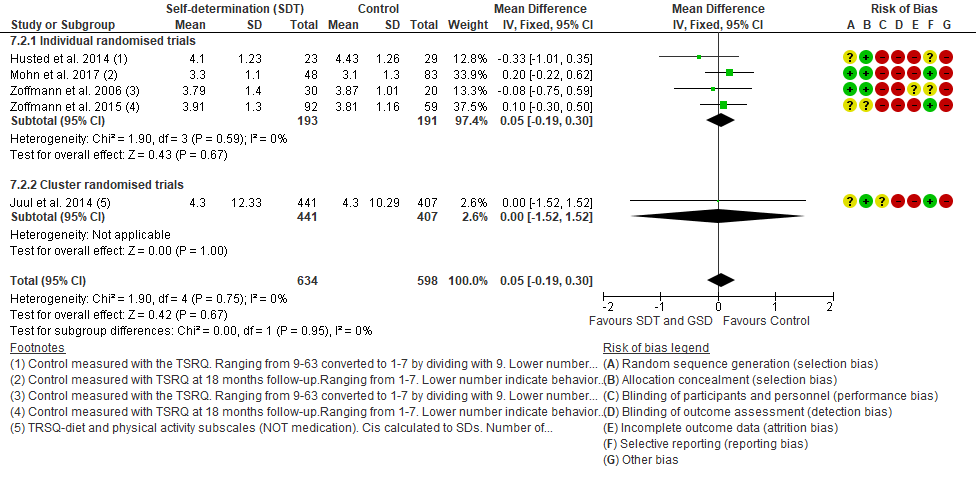
**

**b**


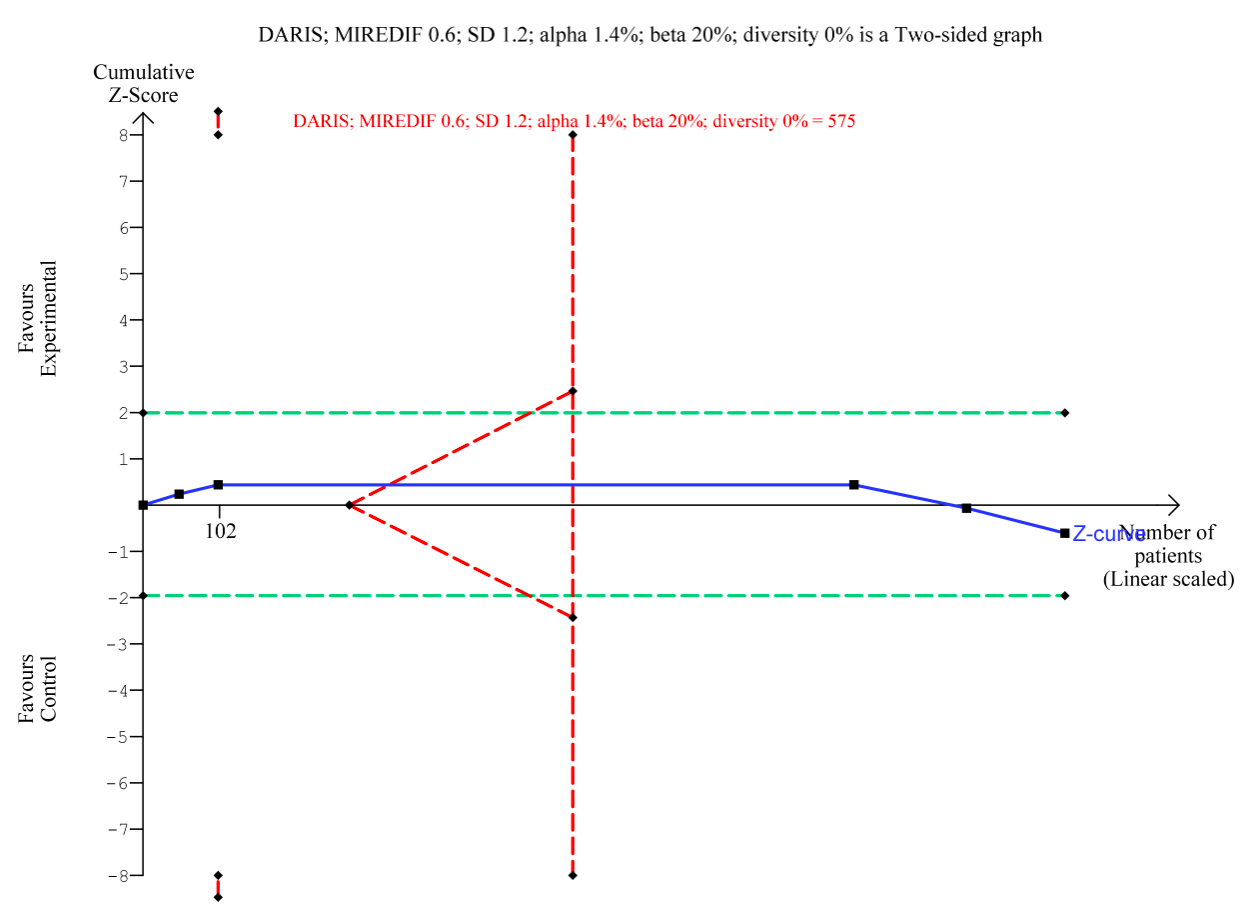
  *Figure 12. Meta-analysis and Trial Sequential Analysis (TSA) for motivation (control), longest follow-up for self-determination theory vs. control.* ***a*** *Meta-analysis.* ***b*** *TSA The diversity adjusted required information size (DARIS) was calculated according to a mean difference of 0.6 points, which is half of the observed SD of 1.2 points, alpha of 1.4%; a beta of 20% (80% power); and diversity 0%. The DARIS was 575 participants. The cumulative Z-curve (blue line) breaches the boundary of futility (dotted outward sloping red lines) and the DARIS. The green dotted lines show naive conventional boundaries (alpha 5%)*

Figure 13. Meta-analysis and Trial Sequential Analysis of motivation (amotivation), end of intervention

**a**

**
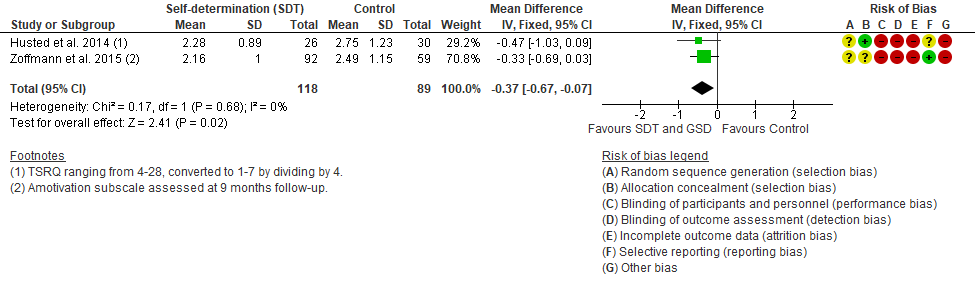
**

**b**


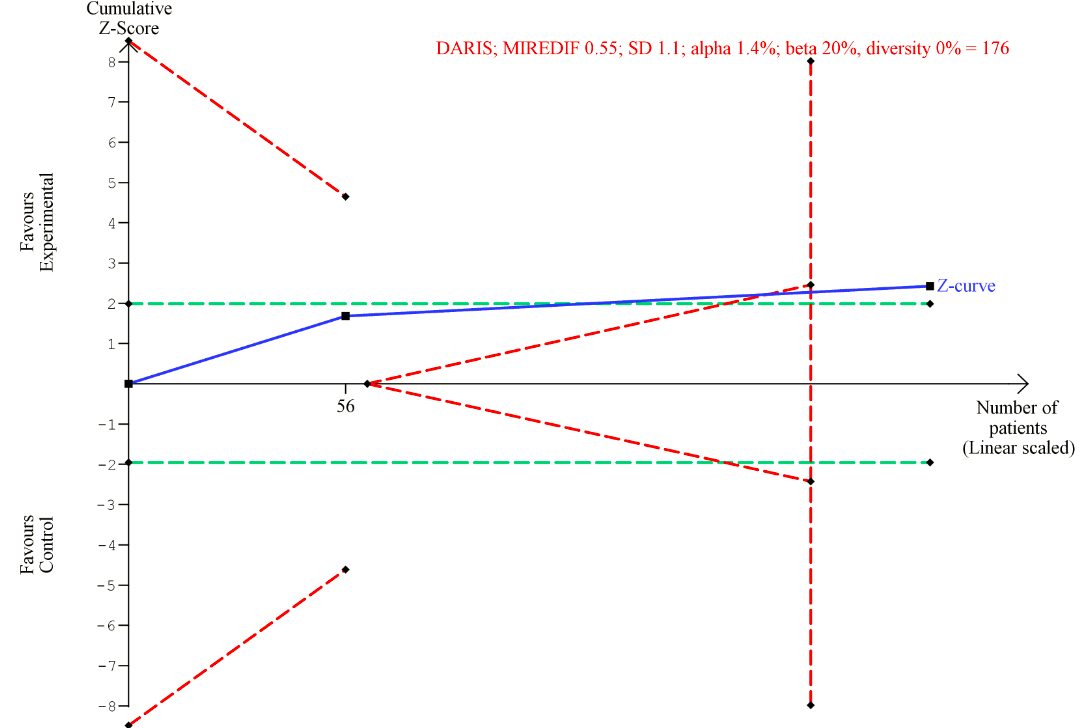


*Figure 13. Meta-analysis and Trial Sequential Analysis (TSA) for motivation (amotivation), end of intervention for self-determination theory vs. control.* ***a*** *Meta-analysis.* ***b*** *TSA The diversity adjusted required information size (DARIS) was calculated according to a mean difference of 0.55 points, which is half of the observed SD of 1.1 points, alpha of 1.4%; a beta of 20% (80% power); and diversity 0%. The DARIS was 176 participants. The cumulative Z-curve (blue line) breaches into futility before the DARIS is reached. The green dotted lines show naive conventional boundaries (alpha 5%).*

Figure 14. Meta-analysis and Trial Sequential Analysis of motivation (amotivation), longest follow-up

**a**

**
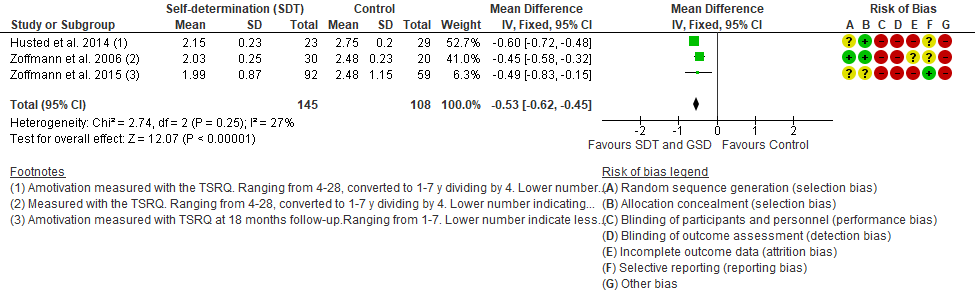
**

**b**


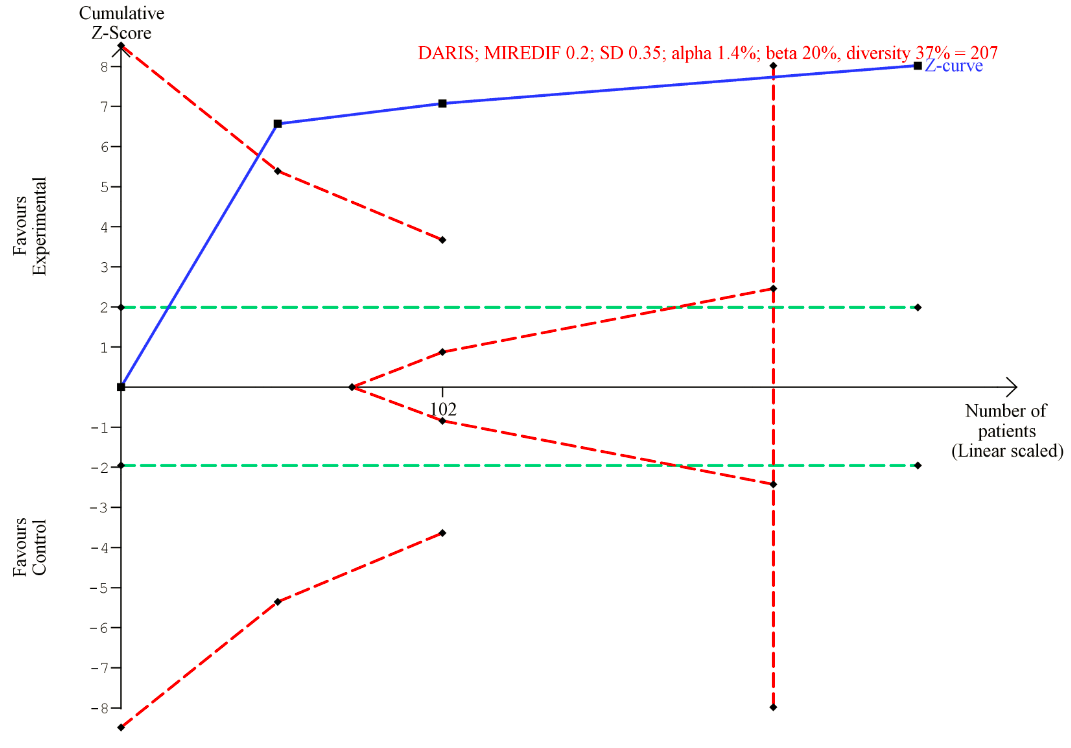


*Figure 14. Meta-analysis and Trial Sequential Analysis (TSA) for motivation (amotivation), longest follow-up for self-determination theory vs. control.* ***a*** *Meta-analysis.* ***b*** *TSA The diversity-adjusted required information size (DARIS) was calculated according to a mean difference of 0.2 points, which is half of the observed SD 0.35 points, alpha of 1.4%; a beta of 20% (80% power); and diversity 37%. The DARIS was 207 participants. The cumulative Z-curve (blue line) breaches the trial sequential monitoring boundary for benefit. The green dotted lines show naive conventional boundaries (alpha 5%).*

1. Mathiesen, A.S., *Vulnerable people with type 2 diabetes: Implications and feasibility of a guided self-determination intervention for reducing diabetes distress*, in *Department of Public health, Faculty of health and Medical Sciences, University of Copenhagen*. 2019: Faculty of Public health, University of Copenhagen.

2. Glasgow, R.E., et al., *Randomized effectiveness trial of a computer-assisted intervention to improve diabetes care.* Diabetes Care, 2005. **28**(1): p. 33-9.
