# Supplementary material for: Self-determination theory interventions versus usual care in people with diabetes: a systematic review with meta-analysis and trial sequential analysis
Source: Syst Rev. 2023 Sep 6;12:158. doi: 10.1186/s13643-023-02308-z (PMC10483731; doi:10.1186/s13643-023-02308-z)
Supplement: Supplementary file 5 — Additional file 5. Serious adverse events (SAE) and Adverse events. [file 13643_2023_2308_MOESM5_ESM.docx]

**Supplementary file 5: serious adverse events (SAE) and Adverse events.**

| **Serious adverse events, longest follow-up** |  |  |
| --- | --- | --- |
| **Trial** | **Intervention group** | **Control group** |
| **Brorson et al. (2019)^1^** |  | 1 participant were excluded from the control group due to extremely high values of HbA1c at 12 months (13.6%, 125 mmol/mol) |
| **Juul et al. (2014)** | 42 participants died (register data) | 38 participants died (register data) |
|  |  |  |
|  |  |  |
|  |  |  |
|  |  |  |
| 1. reported in the publication, not a pre-specified outcome | |  |

| **Serious adverse events, end of intervention** |  |  |
| --- | --- | --- |
| **Trial** | **Intervention group** | **Control group** |
| **Brorson et al. (2019)^1^** |  | 1 participant were excluded from the control group due to extremely high values of HbA1c 6 months (12.5%, 113 mmol/mol) |
| **Mohn et al. (2017)** | 1 person dropping out due to referral to "psychiatric care" mentioned as drop-out in the experimental group and "other critical disease" as drop-out in the control group. | 1 person dropping out due to "other critical disease". |
|  |  |  |
| 1. reported in the publication, not a pre-specified outcome | |  |
|  | |  |

.

| **Adverse events (non-serious), end of intervention** |  |  |
| --- | --- | --- |
| **Trial** | **Intervention group** | **Control group** |
| **Mathiesen (2019)^1^** | 1 person relapsing in psychiatric illness (paranoid schizofrenia) | None reported |
|  |  |  |
|  |  |  |
|  |  |  |
|  |  |  |
| 1) reported in the publication, not a pre-specified outcome | |  |
