# Supplementary material for: Self-determination theory interventions versus usual care in people with diabetes: a systematic review with meta-analysis and trial sequential analysis
Source: Syst Rev. 2023 Sep 6;12:158. doi: 10.1186/s13643-023-02308-z (PMC10483731; doi:10.1186/s13643-023-02308-z)
Supplement: Supplementary file 6 — Additional file 6. Subgroup analyses, Quality of life. [file 13643_2023_2308_MOESM6_ESM.docx]

**Supplementary file 6: Subgroup analyses, Quality of life**

When assessing quality of life, test for subgroup difference showed no evidence of a difference when comparing types of diabetes (*p*=0.17), sex (men compared to women) (*p*=0.12), age (adolescents compared to adults (*p*=0.12), length of intervention (trials with intervention length below compared to above 226 days (the average intervention length))(*p*=0.14), type of therapy (individual compared to group) (*p*=0.06), or type of control intervention (standard care compared to waitlist design compared to attention control) (*p*=0.24).

We were not able to perform any of the remaining pre-planned subgroup analyses [1] due to lack of relevant data.

**Participants:**

1. Type of diabetes: Type 1 diabetes compared to type 2 diabetes

Primary outcome: Quality of life


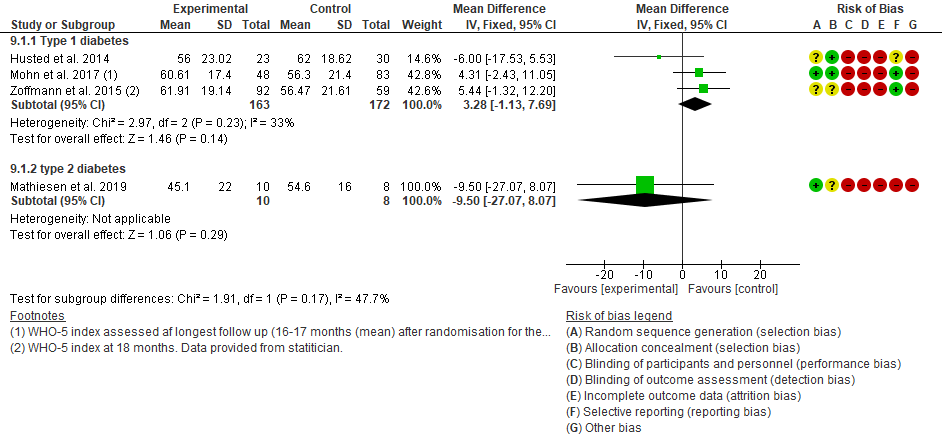


1. Socioeconomic status (Low vs. High socioeconomic status). Not possible to conduct due to sparse data.
2. Number of comorbidities. Not possible to conduct due to sparse data.
3. Effect in men compared to women.


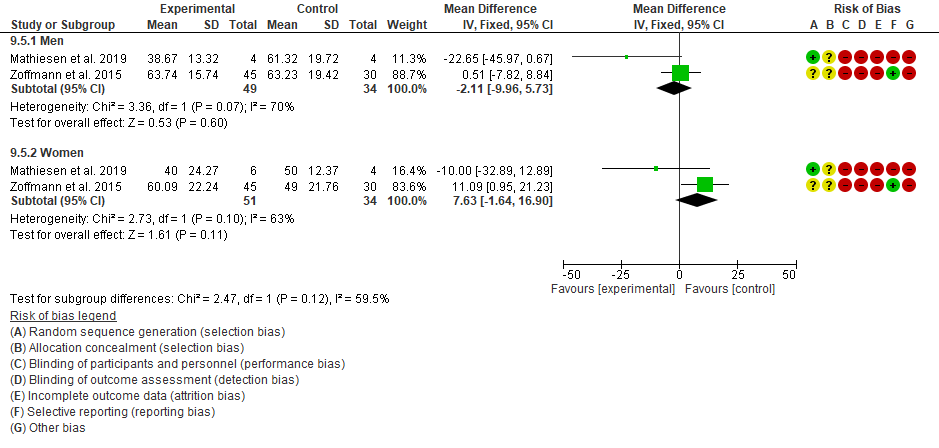


1. Effect in adolescents (13-18 years) compared to adults (> 18 years);


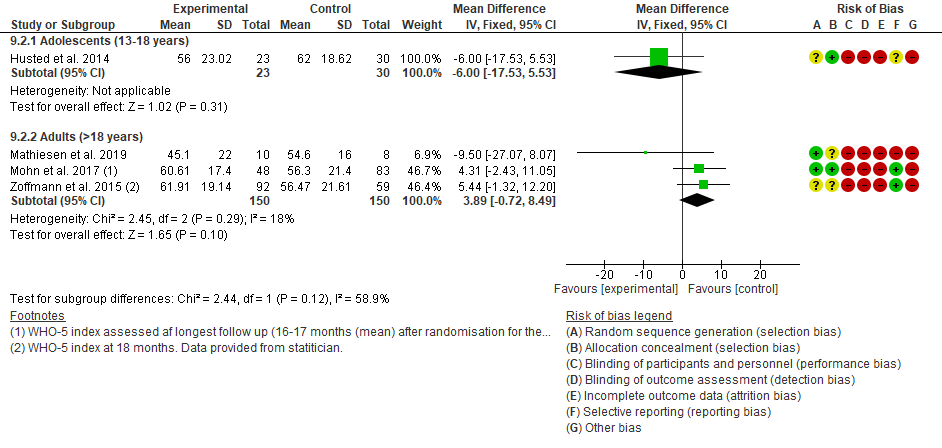


**Intervention:**

6. Trials investigating self-determination theory-based interventions compared to guided self-determination method.

Only trials investigating guided self-determination interventions reported on Quality of life.

7. Trials with interventions above compared to below the mean difference in intervention length. The mean difference of the four trials reporting on quality of life was 228 days (range 98-608).


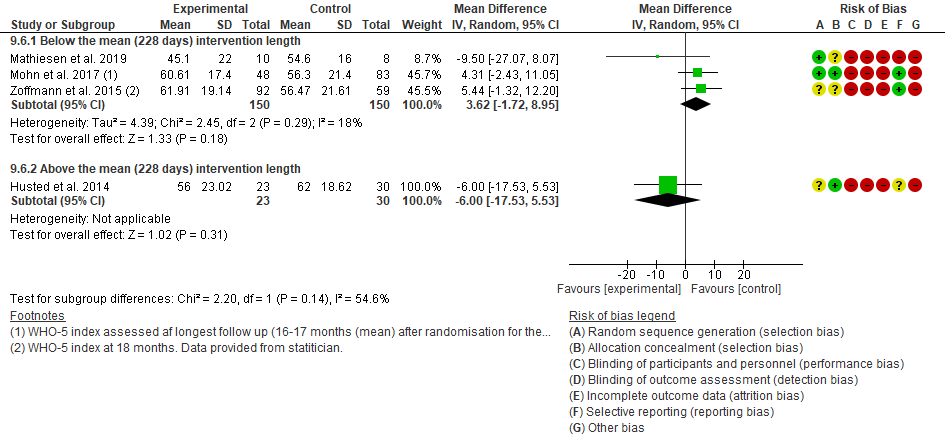


8. Individual compared to group interventions; Quality of life


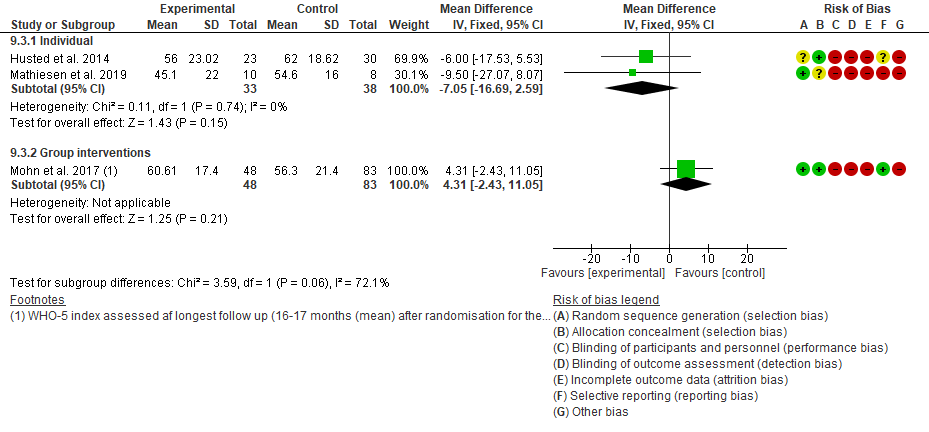


9. Type of control intervention (no intervention compared to standard care compared to placebo attention control).


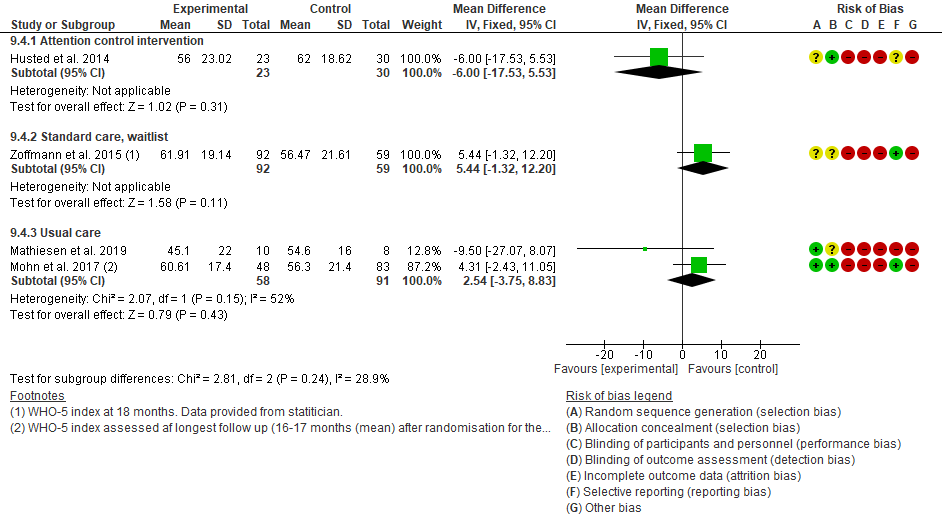


**Risk of bias**

10. Trials at low risk of bias compared to trials at high risk of bias

Not possible to conduct due to the inclusion of high risk of bias trials only.

1. Mathiesen, A.S., et al., *Self-determination theory interventions versus usual care in people with diabetes: a protocol for a systematic review with meta-analysis and trial sequential analysis.* Syst Rev, 2021. **10**(1): p. 12.
