# Supplementary material for: Self-determination theory interventions versus usual care in people with diabetes: a systematic review with meta-analysis and trial sequential analysis
Source: Syst Rev. 2023 Sep 6;12:158. doi: 10.1186/s13643-023-02308-z (PMC10483731; doi:10.1186/s13643-023-02308-z)

**Supplementary file 7: Subgroup analyses, Diabetes distress**

When assessing diabetes distress, test for subgroup difference showed evidence of a difference when comparing type of diabetes (*p*=0.02) with a neutral effect of the experimental intervention in participants with type 1 diabetes and a negative effect in participants with type 2 diabetes; Guided self-determination method compared to self-determination theory-based interventions (*p*=0.007) showing benefits of guided self-determination and harms of self-determination based interventions; type of therapy (individual compared to group (*p*=0.004) showing harms of individual therapy compared to benefits of group therapy. We found no evidence of a difference when comparing adolescents to adults (*p*=0.15), length of intervention (*p*=0.14), or type of control intervention (standard care compared to waitlist design compared to attention control) (*p*=0.05).

We were not able to perform any of the remaining pre-planned subgroup analyses [1] due to lack of relevant data.

**Participants:**

1. Type of diabetes: Type 1 diabetes compared to type 2 diabetes

Primary outcome: Diabetes distress


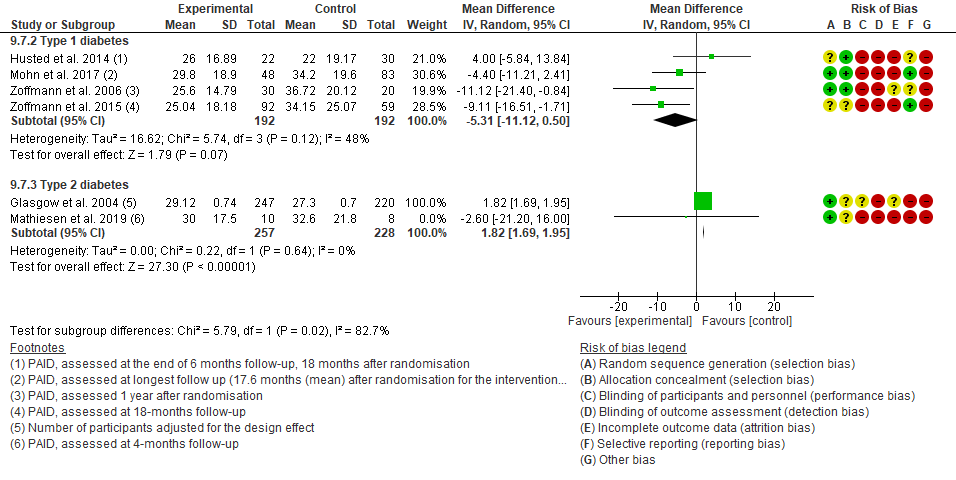


1. Socioeconomic status (Low compared to high socioeconomic status). Not possible to conduct due to sparse data.
2. Number of comorbidities. Not possible to conduct due to sparse data.
3. Effect in men compared to women. Not possible to conduct due to sparse data.
4. Effect in adolescents (13-18 years) compared to adults (> 18 years):


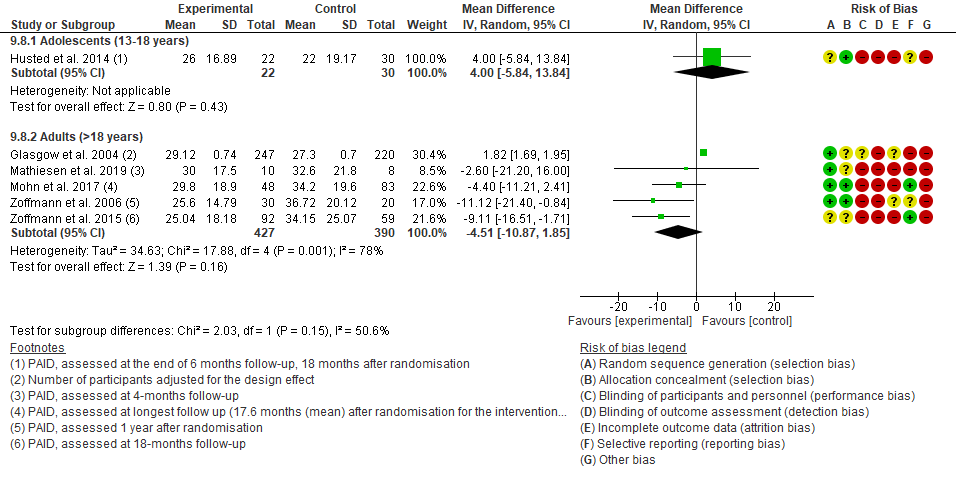


**Intervention:**

6. Trials investigating self-determination theory-based interventions compared to guided self-determination method.


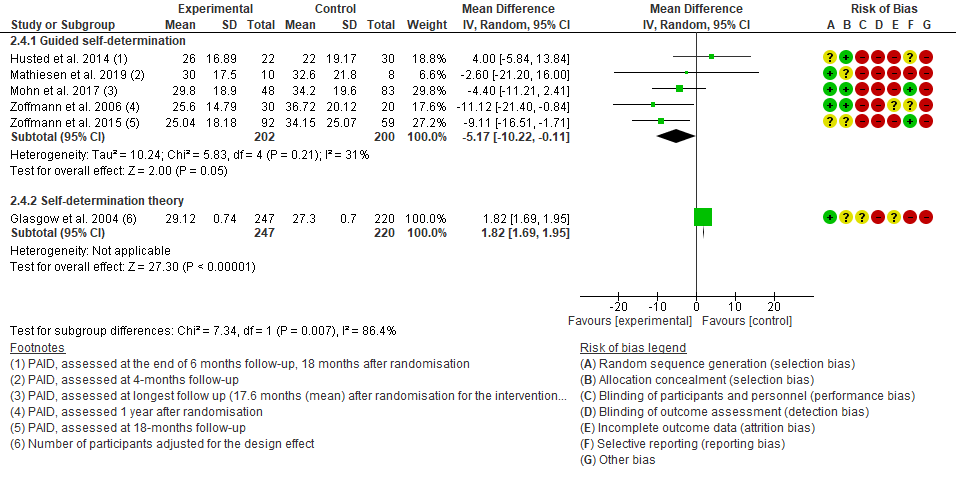


1. 7. Interventions above or below the mean difference in intervention length. The mean difference of the four trials reporting on quality of life was 228 days (range 98-608). Not possible to conduct due to sparse data.

8. Individual compared to group interventions:


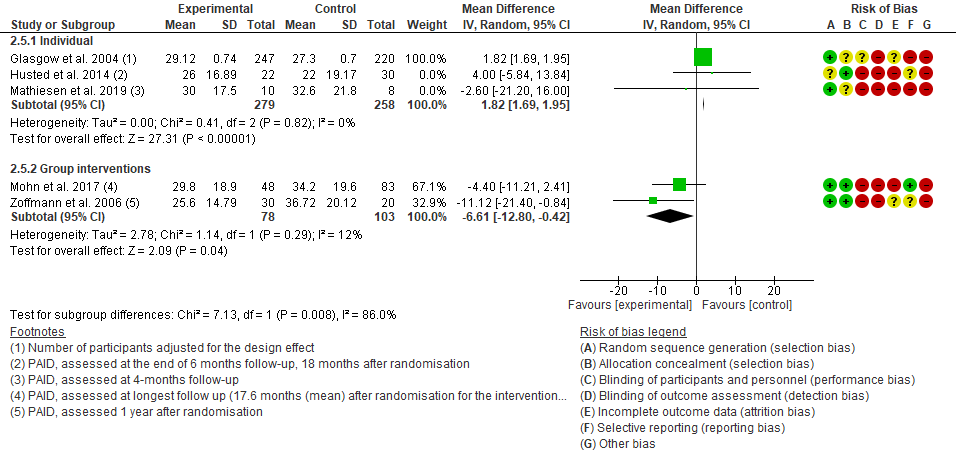

Supplement: Supplementary file 7 — Additional file 7. Subgroup analyses, Diabetes distress. [file 13643_2023_2308_MOESM7_ESM.docx]
